# Tackling clinical heterogeneity across the amyotrophic lateral sclerosis–frontotemporal dementia spectrum using a transdiagnostic approach

**DOI:** 10.1093/braincomms/fcab257

**Published:** 2021-10-23

**Authors:** Rebekah M Ahmed, Martina Bocchetta, Emily G Todd, Nga Yan Tse, Emma M Devenney, Sicong Tu, Jashelle Caga, John R Hodges, Glenda M Halliday, Muireann Irish, Matthew C Kiernan, Olivier Piguet, Jonathan D Rohrer

**Affiliations:** 1 Memory and Cognition Clinic, Institute of Clinical Neurosciences, Royal Prince Alfred Hospital, Sydney 2050, Australia; 2 Brain and Mind Centre, The University of Sydney, Sydney, NSW 2050, Australia; 3 Dementia Research Centre, Department of Neurodegenerative Disease, UCL Queen Square Institute of Neurology, University College London, London WC1E, UK; 4 School of Psychology and Brain and Mind Centre, The University of Sydney, Sydney 2050, Australia

**Keywords:** Frontotemporal dementia-amyotrophic lateral sclerosis, behavioural, cognition, imaging

## Abstract

The disease syndromes of amyotrophic lateral sclerosis (ALS) and frontotemporal dementia (FTD) display considerable clinical, genetic and pathological overlap, yet mounting evidence indicates substantial differences in progression and survival. To date, there has been limited examination of how profiles of brain atrophy might differ between clinical phenotypes. Here, we address this longstanding gap in the literature by assessing cortical and subcortical grey and white matter volumes on structural MRI in a large cohort of 209 participants. Cognitive and behavioural changes were assessed using the Addenbrooke’s Cognitive Examination and the Cambridge Behavioural Inventory. Relative to 58 controls, behavioural variant FTD (*n *=* *58) and ALS–FTD (*n *=* *41) patients displayed extensive atrophy of frontoinsular, cingulate, temporal and motor cortices, with marked subcortical atrophy targeting the hippocampus, amygdala, thalamus and striatum, with atrophy further extended to the brainstem, pons and cerebellum in the latter group. At the other end of the spectrum, pure-ALS patients (*n *=* *52) displayed considerable frontoparietal atrophy, including right insular and motor cortices and pons and brainstem regions. Subcortical regions included the bilateral pallidum and putamen, but to a lesser degree than in the ALS–FTD and behavioural variant FTD groups. Across the spectrum the most affected region in all three groups was the insula, and specifically the anterior part (76–90% lower than controls). Direct comparison of the patient groups revealed disproportionate temporal atrophy and widespread subcortical involvement in ALS–FTD relative to pure-ALS. In contrast, pure-ALS displayed significantly greater parietal atrophy. Both behavioural variant FTD and ALS–FTD were characterized by volume decrease in the frontal lobes relative to pure-ALS. The motor cortex and insula emerged as differentiating structures between clinical syndromes, with bilateral motor cortex atrophy more pronounced in ALS–FTD compared with pure-ALS, and greater left motor cortex and insula atrophy relative to behavioural variant FTD. Taking a transdiagnostic approach, we found significant associations between abnormal behaviour and volume loss in a predominantly frontoinsular network involving the amygdala, striatum and thalamus. Our findings demonstrate the presence of distinct atrophy profiles across the ALS–FTD spectrum, with key structures including the motor cortex and insula. Notably, our results point to subcortical involvement in the origin of behavioural disturbances, potentially accounting for the marked phenotypic variability typically observed across the spectrum.

## Teaser

Ahmed et al.’s findings demonstrate the presence of distinct atrophy profiles across the ALS–FTD spectrum, with key structures including the motor cortex and insula. Subcortical involvement is likely the origin of behavioural disturbances, potentially accounting for the marked phenotypic variability typically observed across the ALS-FTD spectrum.

## Graphical Abstract

**Graphical Abstract:**
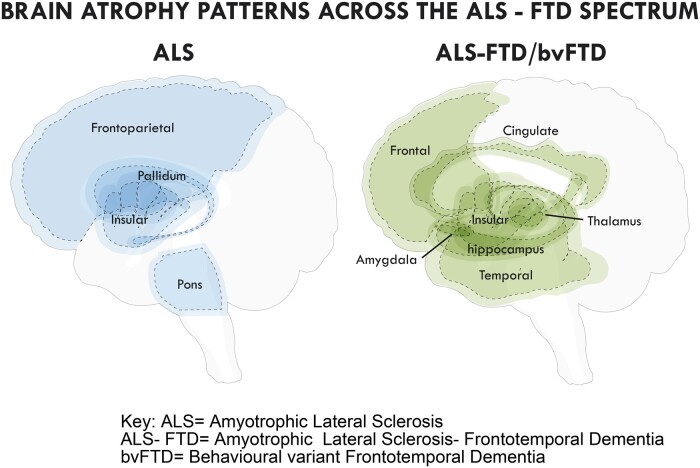

## Introduction

Due to the considerable overlap between amyotrophic lateral sclerosis (ALS) and frontotemporal dementia (FTD) at the clinical, neuropathological and genetic levels, these disorders are posited to lie on a disease spectrum^1^ where ALS represents a predominantly motor phenotype, FTD a cognitive/behavioural phenotype, and ALS–FTD is situated somewhere in between.^2^^,^^3^ Approximately 15% of ALS patients satisfy the diagnostic criteria of concomitant FTD,^4^ and conversely, 10–15% of FTD patients develop ALS, while 25–30% present with motor neuron dysfunction not reaching criteria for ALS.^2^^,^^5^ While a proportion of these clinical syndromes share the *C9orf72* gene expansion^6^^,^^7^ as well as TDP-43 protein deposition in the brain at post-mortem,^8–10^ patients are clinically classified depending on their variable initial profiles of cognitive, behavioural and/or motor disturbances.^11^ Despite these commonalities in genetic mutations and underlying pathology, pathological studies confirm that up to 50% of FTD cases can have underlying Tau pathology.^12^ Currently, apart from those patients harbouring a *C9orf72* repeat expansion, where we know the pathology is likely to be TDP-43, there is no proven method of reliably identifying the likely underlying pathology *in vivo*. Rather, patients are classified along the ALS–FTD spectrum based on their clinical features at presentation. Delineating the neuroanatomical signatures and potential differences between these clinical phenotypes therefore offers an opportunity to refine our understanding of possible underlying disease mechanisms during life. 

The phenotypic motor changes in ALS have been proposed to arise from degeneration primarily targeting the motor neocortex, progressing to the spinal cord and brainstem, which gradually encroaches into frontoparietal and temporal cortices with increasing disease severity.^10^^,^^13^^,^^14^ This spread of atrophy from regions supporting motor function to those implicated in higher-order cognitive processes account for the emergence of cognitive symptoms such as language, executive and memory dysfunction in ALS.^15–17^ By contrast, atrophy in FTD initially targets frontoinsular cortices,^18^ encroaching into adjacent prefrontal and lateral temporal regions, subcortical regions and eventually into the motor and visual cortices,^19^ producing a cluster of behavioural, cognitive and ultimately motor features.

Only a handful of studies have explored the neural correlates of phenotypic profiles across the ALS–FTD spectrum, with the majority of studies constraining their focus to either ALS or FTD. Moreover, of those studies incorporating neuroimaging data, samples sizes have been relatively small, limiting the capacity to detect meaningful brain–behavioural relationships. Previous imaging studies have suggested that prefrontal atrophy is a marker of behavioural-variant FTD (bvFTD) compared with ALS, while greater temporal lobe atrophy potentially differentiated ALS–FTD from ALS.^20^ The extent of motor cortex atrophy in these syndromes remains unclear, with some studies suggesting anterior cingulate and motor cortex degeneration in ALS,^20^ while others using visual rating scales indicating greater motor cortex atrophy in ALS–FTD relative to ALS.^21^ Meta analyses of grey matter atrophy in ALS have suggested involvement of the frontal, temporal and somatosensory regions.^22^ How such varying profiles of atrophy relate to the diversity of cognitive and behavioural changes across the ALS–FTD spectrum remains unknown.

To our knowledge, no large-scale study has combined fine-grained clinical phenotyping with neuroimaging to comprehensively chart the unfolding of symptoms and their neural bases across the ALS–FTD spectrum. As such, this study aimed to provide a detailed characterization of cortical and subcortical atrophy patterns in a large cohort of patients across the ALS–FTD spectrum, defined by clinical presentation. Patients were included in the study based on their clinical presentation, bvFTD, ALS–FTD and pure ALS, rather than their underlying genetic mutation status or presumed pathology in order to provide a clinically relevant sample in which to measure brain atrophy patterns at presentation. Specifically, we aimed to determine phenotypic patterns of cortical and subcortical atrophy at initial presentation, and their relationship with canonical cognitive and behavioural disturbances in ALS and FTD. In doing so, we aimed to develop a refined understanding of the underlying neural mechanisms that give rise to distinct cognitive and behavioural manifestations across the ALS–FTD spectrum with a view to improving the diagnosis and management of these patients.

## Materials and Methods

### Participants

A total of 209 participants between 2009 and 2018 were recruited, 99 individuals diagnosed with bvFTD (*n *=* *58) or ALS–FTD (*n *=* *41) were recruited from the FRONTIER clinic, the multidisciplinary clinical research clinic specializing in FTD and related younger-onset dementias. A further 52 ALS patients were recruited from the multidisciplinary FOREFRONT ALS and FTD clinic, specializing in the diagnosis and management of motor neurodegenerative syndromes. Both clinics are based at the Brain and Mind Centre at The University of Sydney, Australia. Patients were included in each diagnostic group based on their phenotypic presentation, rather than family history or genetic status. Diagnostic assessment consisted of a medical and neurological examination, comprehensive neuropsychological assessment, clinical interviews and a structural brain MRI. Functional assessment in the ALS and ALS–FTD patients at initial presentation was measured using the revised ALS functional rating scale (ALSFRS-R).^23^^,^^24^ Diagnosis was determined by multidisciplinary consensus by a senior neurologist, clinical neurophysiologist and clinical neuropsychologist in accordance with current clinical diagnostic criteria.^25–27^ ALS patients were classified as pure ALS (no cognitive changes) or ALS–FTD. Patients with ALS with cognitive or behavioural impairment that did not meet criteria for ALS–FTD were not included. Fifty-eight healthy participants matched for age and education were included as controls. Inclusion criteria for controls required a score above the cut-off for normal range (>88/100) on the third edition of the Addenbrooke’s Cognitive Examination (ACE-III^28^), to ensure the absence of significant cognitive impairment. Exclusion criteria for all participants included the presence of other dementia syndrome and/or psychiatric disorders. All patients underwent screening for the *C9orf72*, granulin and *MAPT* mutations, and *SOD-1* mutation if an ALS patient. Disease duration was defined as the time between date of symptom onset and date of MRI acquisition.

### Ethics approval

This study was approved by the South Eastern Sydney Local Health District and the University of New South Wales and University of Sydney ethics committees. All the participants or their person responsible provided written, informed consent in accordance with the Declaration of Helsinki.

### Cognitive and behavioural measures

All cognitive and behavioural measures were completed within 3 months of MRI acquisition. Participants completed the ACE-III, comprising a total score as well as attention, memory, fluency, language and visuospatial skills subdomain scores. The trail making test (TMT^29^) was administered to examine processing speed (Part A Time; TMT-A) and executive function (Part B-A time difference; TMT B-A). The revised Cambridge Behavioural Inventory (CBI-R^30^) was used to determine the severity and nature of behavioural symptoms, comprising a total score, as well as 10 subdomain scores for memory and orientation, everyday skills, self-care skills, abnormal behaviour (i.e. behavioural disinhibition), mood changes, odd beliefs (i.e. delusion and hallucinations), abnormal eating habits, sleep, stereotypic behaviours (i.e. perseverative and ritualistic behaviours) and reduced motivation (i.e. apathy and inertia).

### Imaging

#### Brain imaging acquisition

The bvFTD and ALS–FTD group as well as 40 controls underwent volumetric MRI in a 3T Philips Achieva scanner, and a further 10 controls in a 3T General Electric (GE) scanner (both equipped with a standard 8-channel head coil) to obtain high resolution T1-weighted image series using the following parameters (FTD protocol): matrix 256 × 256, 200 slices, 1 mm^2^ in-plane resolution, slice thickness = 1 mm, echo time = 2.6 ms, repetition time = 5.8 ms and flip angle = 8°. The ALS group and a separate group of control participants (*n *=* *8) were scanned on the 3T GE scanner using the following ALS protocol: matrix 256 × 256, 200 slices, 1 mm^2^ in-plane resolution, slice thickness = 0.5 mm, echo time = 2.6 ms, repetition time = 5.8 ms and flip angle = 8°).

#### Brain volume analyses

Volumetric MRI scans were bias field corrected and whole brain parcellated using the geodesic information flow (GIF) algorithm,^31^ which is based on atlas propagation and label fusion. We combined regions of interest to calculate grey and white matter volumes of the lobes (frontal, temporal, parietal, occipital, insula, cingulate), grey matter volumes of the cortex (orbitofrontal; dorsolateral and ventromedial prefrontal; motor; anterior and posterior insula; temporal pole; dorsolateral and medial temporal; sensory; medial and lateral parietal; and anterior, middle and posterior cingulate) and of the subcortical regions (caudate, nucleus accumbens, amygdala, hippocampus, pallidum, putamen, thalamus, pons and brainstem). We also parcellated the whole cerebellum and the vermis.^32^^,^^33^

Total intracranial volume (TIV) was computed with Statistical Parametric Mapping 12 (SPM12) software version 6217 (Statistical Parametric Mapping, Wellcome Trust Centre for Neuroimaging, London, UK) running under Matlab R2014a (Math Works, Natick, MA, USA^34^).

Stringent visual checks were conducted on all MRI scans and segmentations to ensure suitable quality (i.e. motion, other imaging artefacts, pathology unlikely to be attributed to FTD or ALS and incorrect anatomical labelling). Eight participants (three bvFTD, two controls and three ALS-FTD) were removed due to motion artefact and overinclusion of the temporal lobe and hippocampus.

### Statistical analyses

Data were analysed using SPSS Statistics, version 24.0 (IBM, Armonk, NY). The statistical significance level was set at *P *<* *0.05 for all analyses unless otherwise specified. Kolmogorov–Smirnov tests were run to determine suitability of variables for parametric analyses. One-way analysis of variance (ANOVA) was used to compare demographic (i.e. age and education) and cognitive variables (i.e. ACE total and subdomain scores, TMT-A time and TMT B-A time) across all groups (bvFTD, ALS–FTD, ALS and controls), as well as variables specific to patient groups (i.e. disease duration, CBI-R total and subdomain scores, ALSFRS-R scores) followed by Sidak *posthoc* tests. Categorical variables (i.e. sex) were examined using chi-squared tests.

Multivariate analysis of covariance (MANCOVA) was performed to examine differences in the volume of different brain regions between each clinical syndrome and controls (i.e. bvFTD versus controls; ALS-FTD versus controls and ALS versus controls). Age, TIV and sex were included as covariates to control for their confounding effects on brain volumes (*P *<* *0.05 regarded as significant).

Next, exploratory one-sample independent *t*-test analyses were conducted to examine differences in brain volumes between patients with and without *C9orf72* expansion within bvFTD and ALS–FTD groups separately. This analysis was not performed in the ALS group due to the small number of patients with *C9orf72* abnormality (*n *=* *3).

As the MRI scans were acquired using different acquisition protocols for the patient groups and a subset of controls (see the “Brain imaging acquisition” section), it was important to control for differences in acquisition when directly comparing patient groups. For each of the brain regions, we computed separately the mean volume in controls acquired on the ‘ALS protocol’ (eight participants), and the mean volume in controls acquired on the ‘FTD protocol’ (50 participants). For each of the patients, we then computed the percentage difference from the mean volumes in controls acquired on the same protocol as the patient. These derived values were used in a one-way ANOVA to examine the differences in brain volumes across all clinical syndromes, followed by Sidak *posthoc* tests (*P *<* *0.05 regarded as significant).

Associations between different brain region volumes, and cognitive and behavioural variables were examined using Pearson’s correlation with statistical significance set at a more conservative level of *P *<* *0.01 to control for Type I error.

### Data availability

The data that support the findings of this study are available from the corresponding author on request until 2030.

## Results

### Demographics

No significant group differences were found in education level or age across all groups, however, sex distribution differed in the ALS (a greater distribution of male participants) and control groups (a greater proportion of female participants; both *P *<* *0.001; Table  1). Direct comparison of the patient groups revealed significantly longer disease duration in bvFTD compared with both ALS–FTD (*P *=* *0.001) and ALS (*P *<* *0.001) groups, as well as a significantly smaller proportion of participants with a *C9orf72* gene expansion in the ALS group compared with the bvFTD and ALS–FTD groups (both *P*-values <0.001). There were no differences between the ALS and ALS–FTD groups in terms of limb versus bulbar onset (*P *=* *0.260). Seventeen bvFTD patients, 12 ALS-FTD and 3 ALS patients harboured the *C9orf72* expansion, with the ALS group having a lower proportion than the other patient groups (*P *<* *0.001). No significant difference was present between the ALS–FTD and ALS groups on the ALS-FRS-R score, suggesting the groups were comparable in terms of functional impairment (*P *=* *0.229).

**Table 1:** Demographic characteristics of study participants

	Controls	bvFTD	ALS–FTD	ALS^*^	*F*	*P*	*Post hoc*
	(*n *=* *58)	(*n *=* *58)	(*n *=* *41)	(*n *=* *52)			
Sex (M/F)	25/33	38/20	31/10	42/10	19.961^a^	<0.001	Controls and ALS
Age (years)	63.50(10.79)	61.74(8.32)	64.46(8.25)	60.27(10.73)	1.804	0.148	–
Education (years)	13.43(2.58)	12.44(3.05)	12.70(3.20)	13.02(2.55)	1.206	0.309	–
Disease duration (months)	–	60.98(49.46)	32.93(22.63)	27.97(27.55)	9.078	<0.001	bvFTD > ALS–FTD, ALS
*C9orf72* repeat expansion (present/absent)	–	17/41	12/29	3/49	11.296^a^	0.004	ALS < bvFTD and ALS–FTD
Limb versus bulbar onset		NA	25/16	35/15	2.60^a^	0.260	
ALSFRS-R score (/45)			41	42	2.70^b^	0.229	
ACE-III total (/100)	94.58(3.42)	77.07(15.69)	71.95(14.39)	92.37(5.49)	50.080	<0.001	Controls, ALS > bvFTD, ALS–FTD
ACE-Attention (/18)	17.17(0.91)	14.85(2.85)	15.25(2.61)	17.01(1.36)	16.283	<0.001	Controls, ALS > bvFTD, ALS–FTD
ACE-Memory (/26)	24.68(1.62)	18.98(5.34)	19.00(5.21)	23.23(4.33)	31.387	<0.001	Controls, ALS > bvFTD, ALS–FTD
ACE-Fluency (/14)	12.13(1.62)	6.88(3.97)	5.00(3.68)	11.30(1.83)	62.191	<0.001	Controls, ALS > bvFTD, ALS–FTD
ACE-Language (/26)	25.12(0.94)	22.38(4.31)	19.28(4.64)	24.48(1.97)	26.037	<0.001	Controls, ALS > bvFTD, ALS–FTD
							bvFTD > ALS–FTD
ACE-Visuospatial (/16)	15.50(0.87)	14.00(2.60)	13.65(2.06)	15.49(0.92)	14.099	<0.001	Controls, ALS > bvFTD, ALS–FTD
TMT-A time (seconds)	32.73(10.99)	62.61(58.44)	62.94(28.44)	32.35(10.14)	16.245	<0.001	Controls, ALS > bvFTD, ALS–FTD
TMT-B-A time (seconds)	37.89(19.32)	110.00(103.29)	117.74(86.40)	52.47(42.20)	12.183	<0.001	Controls, ALS > bvFTD, ALS–FTD
CBI-R total	–	65.16(29.20)	44.22(30.87)	25.52(20.42)	26.787	<0.001	bvFTD, ALS–FTD > ALS
							bvFTD > ALS–FTD
Memory	–	43.27(21.39)	34.68(23.78)	12.05(12.93)	41.336	<0.001	bvFTD, ALS–FTD > ALS
Everyday skills	–	30.97(25.92)	20.89(20.38)	18.03(30.02)	3.262	0.042	–
Self-care skills	–	16.27(25.65)	8.45(16.42)	22.73(31.27)	3.509	0.035	–
Mood changes	–	29.63(22.20)	21.28(20.44)	16.90(15.72)	5.228	0.007	bvFTD > ALS
Odd beliefs	–	10.06(17.22)	5.41(12.61)	0.24(1.41)	12.127	<0.001	bvFTD, ALS–FTD > ALS
Abnormal behaviours	–	36.57(23.44)	22.86(22.43)	8.45(12.19)	29.541	<0.001	bvFTD, ALS–FTD > ALS
							bvFTD > ALS–FTD
Eating habits	–	41.59(30.50)	23.65(27.99)	8.58(12.17)	27.793	<0.001	bvFTD, ALS–FTD > ALS
							bvFTD > ALS–FTD
Sleep	–	43.75(25.03)	27.70(26.04)	34.09(27.47)	4.526	0.013	bvFTD > ALS–FTD
Stereotypic and motor behaviours	–	41.45(28.91)	34.63(30.68)	8.64(12.69)	33.322	<0.001	bvFTD, ALS–FTD > ALS
Reduced motivation	–	58.88(30.06)	34.77(29.00)	13.57(17.34)	42.559	<0.001	bvFTD, ALS–FTD > ALS
							bvFTD > ALS–FTD

Means (standard deviation).

a Chi-square value.

b *t*-test value.

* 4 left hand, 48 right hand.

#### Cognitive profiles

Both bvFTD and ALS–FTD demonstrated significantly lower overall cognitive performance on the ACE-III total (both *P *<* *0.001), and across all subscales (all *P*-values ≤0.001) relative to controls, with no significant impairments evident in the ALS group (all *p*-values > 0.05). Behavioural variant FTD and ALS–FTD further displayed reduced processing speed on the TMT-A (*P *=* *0.002 and *P *<* *0.001, respectively) and executive dysfunction on the TMT-B-A (*P *<* *0.001 and *P *=* *0.004, respectively). A similar profile of cognitive impairment was observed in bvFTD and ALS–FTD relative to the ALS group, whereby ALS patients outperformed the bvFTD and ALS–FTD groups in terms of overall cognitive function (ACE-III total; both *P*-values <0.001), all subdomains on the ACE-III (all *P*-values <0.01, see Table  1), processing speed and executive function on the TMT measures (all *P*-values <0.025). Taken together, these findings reveal generalized cognitive impairment in bvFTD and ALS–FTD compared with ALS patients and healthy controls. Interestingly, the ALS–FTD group showed greater impairment on the ACE-III language subdomain score compared with bvFTD patients (*P *=* *0.008).

#### Behavioural profiles

**Figure 1:**
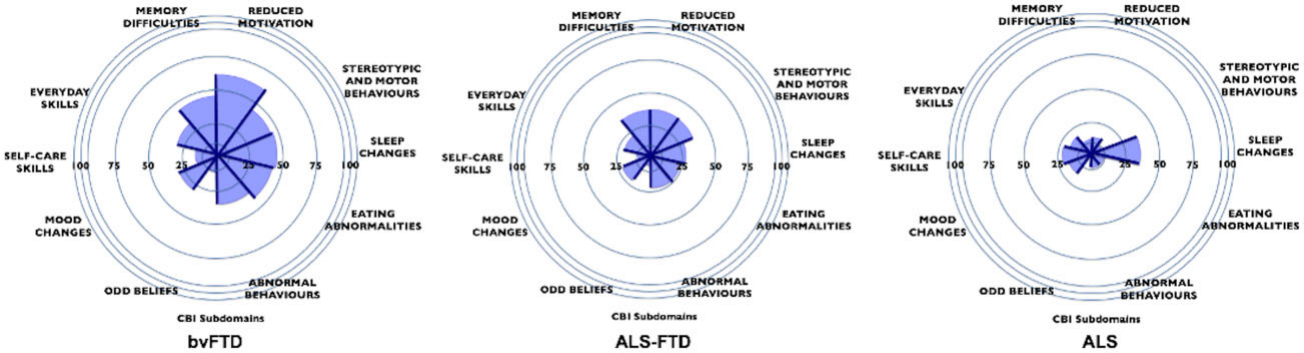
**Severity of behavioural disturbance on all 10 CBI-R subdomains across the ALS–FTD spectrum.** Segments represent mean percentage scores. The longer the segment, the more severe the behavioural disturbance.

Figure  1 displays the behavioural changes across groups. Relative to the ALS group, both bvFTD and ALS–FTD showed significantly greater behavioural disturbances (CBI-R total score: *P *<* *0.001 and *P *=* *0.012). Looking across the CBI-R subscales, bvFTD and ALS-FTD exhibited pervasive behavioural disturbances in memory (both *P*-values <0.001), odd beliefs (*P *<* *0.001 and *P *=* *0.046), abnormal behaviours (*P *<* *0.001 and *P *=* *0.003), eating habits (*P *<* *0.001 and *P *=* *0.012), stereotypic and motor behaviours (both *P*-values <0.001) and reduced motivation (*P *<* *0.001 and *P *=* *0.001) relative to the ALS group. Disproportionate impairments were evident in bvFTD relative to ALS–FTD in terms of overall behavioural disturbances (CBI-R total; *P *=* *0.004) including abnormal behaviours (*P *=* *0.015), eating habits (*P *=* *0.012), sleep changes (*P *=* *0.012) and reduced motivation (*P *=* *0.001), as well as mood changes (*P *=* *0.005) compared with ALS. These findings indicate a graded variation in behavioural disturbances across the ALS–FTD spectrum, with most pronounced behavioural abnormalities emerging in the bvFTD group.

### Imaging results

#### Patterns of volumetric differences in each clinical syndrome relative to controls

**Figure 2:**
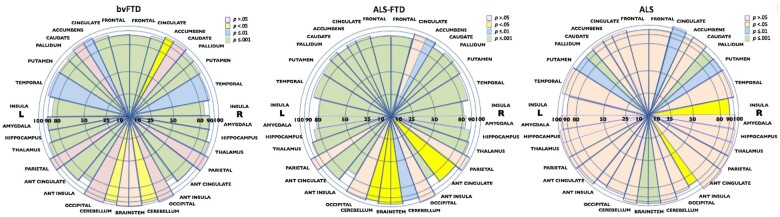
**Percentage difference in regional volumes from controls across the ALS–FTD spectrum.** The relative size of each brain region is represented by the size of the segment (i.e. the larger the segment, the larger the volume of the brain region relative to TIV). The colours denote the different *P*-values from the multivariate analysis of covariance (MANCOVA) examining the difference in volume of different brain regions between each clinical syndrome and controls (i.e. bvFTD versus controls; ALS-FTD versus controls and ALS versus controls) with age, TIV and sex included as covariates. L = Left; R = Right.

When compared with protocol-matched controls the cognitive end of the spectrum showed more extensive frontal and subcortical atrophy. Specifically, bvFTD patients demonstrated significantly lower cortical and subcortical volumes largely concentrated in the bilateral frontal (including motor), limbic (both cingulate and insular) and temporal cortices and subcortical structures including bilateral hippocampus, amygdala, putamen, pallidum and thalamus, with relative sparing of the parietal and occipital cortices (Fig.  2 and Supplementary Table 1). ALS–FTD patients exhibited atrophy involving the bilateral frontal (including motor), limbic (insular and left anterior cingulate) and temporal cortices, as well as subcortical structures including bilateral hippocampus, amygdala, striatum, thalamus, pons and right cerebellum (Supplementary Table 2). In ALS, lower cortical and subcortical volumes were found predominantly in right motor, insula and bilateral prefrontal and parietal (including sensory and lateral parietal) cortices, as well as bilateral putamen, pallidum, pons and brainstem (Supplementary Table 3). The region impacted most severely across all three groups was the insula, and specifically the anterior part (76–90% of control values controls).

### Volumetric differences across ALS–FTD clinical syndromes

#### Cortical atrophy

Direct comparisons between the patient groups revealed significantly different profiles of cortical atrophy. The ALS group displayed greater posterior atrophy, specifically of the bilateral lateral parietal, sensory and occipital cortex volumes compared with both bvFTD and ALS–FTD. In contrast, bvFTD and ALS–FTD showed greater atrophy across the frontal regions, specifically the anterior cingulate and insular cortices relative to the ALS group (Table  2), with disproportionate atrophy of left orbitofrontal cortex, left temporal and bilateral insula and motor cortex in ALS–FTD relative to ALS. Similarly, ALS–FTD displayed greater atrophy of the left motor cortex and left insula relative to the bvFTD group.

**Table 2:** Volumetric percentage of control difference between patient groups^a^

Brain region	bvFTD	ALS–FTD	ALS	*F*	*P*	*Post hoc*	*P*
	(*n *=* *58)	(*n *=* *41)	(*n *=* *52)				
Total frontal lobe	91.04(8.09)	89.15(5.84)	95.31(6.00)	10.218	<0.001	ALS > bvFTD	0.004
						ALS > ALS–FTD	<0.001
Left	91.41(8.79)	89.17(5.52)	95.12(5.96)	8.515	<0.001	ALS > bvFTD	0.02
						ALS > ALS–FTD	<0.001
Right	90.68(8.90)	89.13(6.57)	95.50(6.31)	9.634	<0.001	ALS > bvFTD, ALS–FTD	<0.001
Dorsolateral prefrontal cortex							
Left	89.16(10.31)	87.77(5.97)	90.62(7.18)	1.37	0.257	–	–
Right	89.19(11.13)	89.08(6.78)	92.39(7.05)	2.331	0.101	–	–
Ventromedial prefrontal cortex							
Left	90.00(12.13)	88.77(9.02)	89.22(8.77)	0.185	0.831	–	–
Right	87.52(12.29)	87.79(10.36)	88.83(7.16)	0.241	0.786	–	–
Orbitofrontal cortex							
Left	88.93(13.23)	84.14(8.30)	90.02(6.33)	4.373	0.014	ALS > ALS–FTD	0.015
Right	88.80(10.81)	87.19(8.21)	91.47(7.45)	2.681	0.072	–	–
Motor cortex							
Left	93.69(8.33)	88.61(6.42)	96.58(8.95)	9.399	<0.001	bvFTD > ALS–FTD	0.015
						ALS > ALS–FTD	<0.001
Right	92.97(8.47)	89.14(6.86)	95.67(11.21)	5.225	0.006	ALS > ALS–FTD	0.004
Total cingulate lobe	93.22(7.33)	94.49(5.98)	99.80(6.82)	13.888	<0.001	ALS > bvFTD	<0.001
						ALS > ALS–FTD	0.001
Left	93.28(7.51)	93.18(6.09)	97.70(7.34)	6.755	0.002	ALS > bvFTD	0.004
						ALS > ALS–FTD	0.008
Right	93.16(9.06)	95.94(7.98)	102.17(7.40)	16.959	<0.001	ALS > bvFTD	<0.001
						ALS > ALS–FTD	0.001
Anterior cingulate							
Left	91.63(9.81)	90.79(6.75)	97.46(9.10)	8.484	<0.001	ALS > bvFTD	0.002
						ALS > ALS–FTD	0.001
Right	90.59(9.61)	93.95(9.47)	101.79(10.01)	18.289	<0.001	ALS > bvFTD	<0.001
						ALS > ALS–FTD	0.001
Middle cingulate							
Left	95.83(10.21)	95.12(9.56)	97.77(14.55)	0.663	0.517	–	–
Right	93.74(10.70)	96.02(11.11)	99.74(11.48)	4.049	0.019	ALS > bvFTD	0.014
Posterior cingulate							
Left	96.65(9.36)	98.05(8.25)	98.18(8.07)	0.52	0.596	–	–
Right	95.03(9.63)	98.89(9.55)	102.87(8.84)	9.664	<0.001	ALS > bvFTD	<0.001
Total insular lobe	85.88(15.47)	80.44(10.59)	90.40(8.91)	7.585	0.001	ALS > ALS–FTD	<0.001
Left	86.06(15.94)	78.65(10.91)	90.26(8.54)	10.047	<0.001	bvFTD > ALS–FTD	0.011
						ALS > ALS–FTD	<0.001
Right	85.70(16.36)	82.20(11.80)	90.54(10.08)	4.679	0.011	ALS > ALS–FTD	0.008
Anterior insula							
Left	82.41(18.40)	76.16(11.69)	89.57(9.48)	10.528	<0.001	ALS > bvFTD	0.023
						ALS > ALS–FTD	<0.001
Right	82.71(19.00)	80.17(13.04)	90.18(10.37)	5.929	0.003	ALS > bvFTD	0.026
						ALS > ALS–FTD	0.005
Posterior insula							
Left	93.48(13.73)	83.69(13.14)	91.63(9.75)	8.118	<0.001	bvFTD > ALS–FTD	<0.001
						ALS > ALS–FTD	0.007
Right	91.66(13.65)	86.25(13.61)	91.26(12.40)	2.342	0.1	–	–
Total parietal lobe	100.50(5.75)	99.82(6.97)	99.05(5.64)	0.788	0.456	–	–
Left	100.30(5.53)	100.10(7.39)	98.82(5.33)	0.929	0.397	–	–
Right	100.45(6.53)	99.54(6.95)	99.28(6.20)	0.482	0.619	–	–
Medial parietal cortex							
Left	99.06(9.83)	102.81(11.38)	96.47(8.70)	4.69	0.011	ALS–FTD > ALS	0.007
Right	99.53(10.49)	101.15(9.63)	101.50(8.92)	0.633	0.532	–	–
Lateral parietal cortex							
Left	97.98(9.99)	98.20(10.58)	90.40(10.84)	9.188	<0.001	bvFTD, ALS–FTD > ALS	0.001
Right	96.62(10.44)	97.18(10.16)	90.12(10.06)	7.43	0.001	bvFTD, ALS–FTD > ALS	0.003
Sensory cortex							
Left	98.68(11.90)	100.07(10.38)	92.75(11.14)	5.892	0.003	bvFTD > ALS	0.018
						ALS–FTD > ALS	0.006
Right	98.98(12.68)	102.36(13.35)	91.42(12.94)	8.977	<0.001	bvFTD > ALS	0.008
						ALS–FTD > ALS	<0.001
Total temporal lobe	94.35(7.33)	93.03(8.15)	97.40(5.63)	4.744	0.010	ALS > ALS–FTD	0.012
Left	92.48(15.09)	87.81(18.72)	98.18(6.02)	6.467	0.002	ALS > ALS–FTD	<0.001
Right	91.98(15.04)	86.25(20.40)	96.76(5.69)	5.696	0.004	ALS > ALS–FTD	<0.001
Dorsolateral temporal cortex							
Left	93.22(8.54)	90.08(9.03)	94.83(6.00)	3.986	0.021	ALS > ALS–FTD	0.015
Right	91.48(9.48)	91.07(9.56)	92.60(9.09)	0.332	0.718	–	–
Medial temporal cortex							
Left	96.32(8.50)	91.93(10.12)	99.58(6.37)	9.237	<0.001	ALS > ALS–FTD	<0.001
						bvFTD > ALS–FTD	0.036
Right	95.17(8.44)	92.53(10.37)	94.99(5.28)	1.383	0.254	–	–
Temporal pole							
Left	90.13(17.60)	82.37(17.76)	92.20(12.35)	4.382	0.014	ALS > ALS–FTD	0.013
Right	86.95(17.53)	85.47(18.37)	88.05(12.26)	0.279	0.757	–	–
Total occipital lobe	100.53(7.07)	100.90(7.05)	97.68(6.63)	2.67	0.073	–	–
Left	99.61(7.42)	101.16(7.41)	92.45(18.94)	6.417	0.002	ALS–FTD, bvFTD > ALS	0.01
Right	101.12(7.16)	101.42(7.69)	92.89(19.52)	6.956	0.001	ALS–FTD > ALS	0.01
						bvFTD > ALS	<0.001
Hippocampus							
Left	90.50(9.39)	86.39(10.76)	98.94(6.61)	24.351	<0.001	ALS > ALS–FTD, bvFTD	<0.001
Right	90.17(10.34)	89.90(11.44)	97.78(6.56)	11.116	<0.001	ALS > ALS–FTD, bvFTD	<0.001
Amygdala							
Left	91.43(13.63)	82.96(13.44)	96.19(7.29)	14.618	<0.001	ALS > ALS–FTD	<0.001
						bvFTD > ALS–FTD	0.002
Right	90.43(13.10)	83.71(12.21)	96.38(7.77)	14.494	<0.001	ALS > bvFTD	0.018
						ALS > ALS–FTD	<0.001
						bvFTD > ALS–FTD	0.011
Caudate							
Left	97.00(14.04)	89.68(13.45)	96.80(10.46)	4.444	0.013	ALS > ALS–FTD	0.027
						bvFTD > ALS–FTD	0.021
Right	97.99(14.74)	90.80(12.78)	99.87(13.14)	5.072	0.007	ALS > ALS–FTD	0.007
						bvFTD > ALS–FTD	0.039
Putamen							
Left	91.86(9.66)	89.22(7.58)	94.78(6.28)	5.509	0.005	ALS > ALS–FTD	0.003
Right	92.88(11.23)	91.50(7.77)	96.08(6.00)	3.435	0.035	ALS > ALS–FTD	0.036
Accumbens							
Left	96.11(8.33)	93.47(9.52)	97.86(5.47)	3.564	0.031	ALS > ALS–FTD	0.023
Right	97.58(9.41)	95.03(10.79)	96.06(5.81)	1.061	0.349	–	–
Pallidum							
Left	94.08(8.70)	93.06(7.11)	94.72(6.34)	0.558	0.574	–	–
Right	94.34(9.80)	93.75(8.57)	94.83(6.15)	0.192	0.825	–	–
Thalamus							
Left	94.00(6.18)	91.41(7.76)	98.49(8.24)	11.181	<0.001	ALS > bvFTD	0.005
						ALS > ALS–FTD	<0.001
Right	94.19(5.83)	92.35(7.70)	98.05(8.75)	7.308	0.001	ALS > bvFTD	0.020
						ALS > ALS-FTD	0.001
Total cerebellum	97.09(6.99)	95.34(7.71)	98.93(7.96)	2.626	0.076	–	–
Left	96.72(7.03)	95.61(7.95)	98.97(8.11)	2.39	0.095	–	–
Right	97.14(7.42)	94.99(7.89)	98.93(8.09)	2.934	0.056	–	–
Vermis	99.82(9.61)	100.05(9.37)	99.86(10.58)	0.007	0.993	–	–
Pons	98.88(11.66)	93.41(9.48)	95.64(7.79)	3.846	0.024	bvFTD > ALS–FTD	0.02
Brainstem	99.87(7.53)	97.21(6.68)	97.80(6.35)	2.114	0.124	–	–

a Values are mean percentages of control subjects (standard deviation).

#### Subcortical atrophy

At the cognitive end of the spectrum more extensive subcortical atrophy was present. Specifically, no significant subcortical atrophy was present in ALS relative to the other patient groups. Both ALS–FTD and bvFTD showed greater atrophy in the bilateral hippocampus, thalamus and right amygdala compared with ALS. ALS–FTD had additional putamen, caudate and left amygdala atrophy compared with ALS, and greater atrophy of the bilateral caudate and amygdala compared with bvFTD (Table  2).

#### Patterns of volumetric differences in patients with versus without *C9orf72* expansion

Compared with those without, bvFTD patients harbouring the *C9orf72* expansion displayed significantly lower volumes in left posterior insula, right parietal lobe, bilateral lateral parietal cortex, bilateral medial temporal cortex and left thalamus (Supplementary Table 4). In ALS–FTD, striatal structures (i.e. bilateral putamen, accumbens and pallidum; Supplementary Table 5), and bilateral occipital cortex, thalamus and hippocampus, and the vermis were implicated.

### Correlations between brain volumes and cognitive dysfunction

Within the entire patient cohort (*n *=* *151), performance on the ACE-III was found to correlate with atrophy in distributed cortical and subcortical regions (Supplementary Table 6). Volumes of bilateral frontal, cingulate, insula and temporal cortices, along with subcortical structures including the caudate, nucleus accumbens, hippocampus, amygdala, pallidum, putamen and thalamus, were all positively correlated with higher ACE-III Total scores, as well as better performance across the memory, fluency, language and visuospatial subdomains (all *P*-values <0.01). A similar set of regions was implicated in attention (ACE-III attention and orientation) and processing speed (TMT-A) with the exception of temporal cortex, amygdala, hippocampus and caudate. In terms of executive function, reduced frontal, parietal and thalamus volumes bilaterally were associated with poorer TMT-B-A performance (all *P*-values < 0.01).

### Correlations between brain volumes and behavioural disturbances

Within the overall patient cohort (*n *=* *151), memory difficulties were negatively correlated with volume of bilateral frontal, insular and temporal regions, as well as subcortical regions including the amygdala, hippocampus and nucleus accumbens. Behavioural disturbances commonly observed across the ALS–FTD spectrum such as odd beliefs (i.e. delusions and hallucinations), stereotypic and ritualistic behaviours and reduced motivation (i.e. apathy) were all negatively correlated with bilateral frontal, insular and cingulate regions as well as the nucleus accumbens, hippocampus, putamen, amygdala and thalamus (see Supplementary Table 7). The same regions were implicated in abnormal behaviours (i.e. behavioural disinhibition) and eating abnormalities with the exception of putamen and amygdala. In addition, odd beliefs were negatively associated with bilateral pallidum volumes, while behavioural disinhibition, stereotypic and ritualistic behaviours, and apathy all correlated with bilateral temporal volumes (all *P*-values <0.01).

## Discussion

The current study provides a comprehensive characterization of the clinical, behavioural and neuroanatomical heterogeneity across the ALS–FTD spectrum in a large cohort of patients. Our objective was to combine fine-grained clinical phenotyping with high-resolution 3D neuroimaging to comprehensively chart the unfolding of symptoms, and their neural bases, across the ALS–FTD spectrum. Overall, our findings underscore the marked heterogeneity in cognitive, behavioural and motor features, independent of the clinical diagnosis conferred at initial presentation, and identify differences in associated regional neurodegeneration. The diversity of the underlying neurodegeneration suggests that ALS, ALS–FTD and bvFTD are not simply the same condition with variability in the severity of regional atrophy, but atrophy in key neural structures differentiates the motor from the cognitive and behavioural syndromes, in particular significant cortical atrophy occurred in bvFTD and ALS–FTD, while brainstem atrophy was severe only in ALS (see Fig.  2), consistent with progressive corticospinal tract degeneration.^35^ Of interest, subcortical bilateral caudate atrophy was severe only in ALS–FTD (see Fig.  2), suggesting more widespread impact on basal ganglia circuits, a feature observed with increasing progression of ALS.^36^

Considering first the neuroanatomical profiles of each clinical phenotype compared with controls, at the FTD end of the spectrum, both bvFTD and ALS–FTD were characterized by extensive atrophy involving frontoinsular, cingulate, temporal and motor cortices. Additional extensive involvement of subcortical structures was present, including the bilateral hippocampus, amygdala, nucleus accumbens, pallidum, putamen and thalamus. Atrophy of these structures has previously been reported in bvFTD and strongly underpins the behavioural and emotion processing deficits seen in this clinical syndrome.^18^^,^^37^^,^^38^ While the neuroanatomical signature of ALS–FTD is less well-characterized, often due to the small patient numbers, previous studies have documented frontal and temporal lobe atrophy.^20^ Our results suggest that this atrophy pattern progresses more rapidly to impact many of the subcortical structures that are vulnerable in bvFTD including the hippocampus, nucleus accumbens, amygdala, pallidum, putamen and thalamus. We suggest that this overlap in frontal and subcortical structures likely drives many of the commonalities in terms of behavioural changes that are reported in bvFTD and ALS–FTD, including changes in eating behaviour, and emotion and reward processing.^39^ Both bvFTD and ALS–FTD also displayed cerebellar atrophy compared with controls, again in keeping with previous studies reporting differential patterns of cerebellar involvement across the spectrum, and its relationship to various cognitive and behavioural changes.^40^^,^^41^ In the current study, compared with controls, atrophy in ALS–FTD extended to involve the brainstem, pons and cerebellum. This finding resonates with a recent study in which significant brainstem involvement was reported in ALS and ALS–FTD, particularly involving the medulla oblongata and pons, presumably indicating involvement of upper motor neuron axons in the descending corticospinal tracts.^35^ As previously suggested,^35^ our data show that brainstem involvement may represent an important biomarker in differentiating ALS and ALS–FTD from bvFTD, and in predicting those patients who may go on to develop motor involvement.

At the ALS end of the spectrum, we uncovered significant atrophy in ALS patients relative to controls centred on bilateral prefrontal and lateral parietal cortex, with right lateralized involvement of the posterior cingulate, motor and insular cortex. Previous emerging literature indicates a cerebral hemispheric dominance, with atrophy predominantly affecting the left motor cortex or dominant cortex in ALS,^42^ at least in the early stages of the disease. Right-sided involvement of the cingulate and insular cortices may indicate early emotional processing difficulties^43^ as atrophy progresses to extend beyond the motor cortices in ALS.^11^^,^^44^^,^^45^ In the current study only 4 of the 52 ALS patients were left-handed, so it is unlikely that handedness played a role in the lateralized involvement, however, further research could examine the role of handedness in cerebral predominance. Further studies will also need to include measures of motor involvement in ALS and patterns of spread, beyond the ALSFRS-R score, in relation to brain atrophy patterns, to determine if lateralized cerebral involvement, such as the right predominance shown in this study may be represented clinically in terms of muscle weakness and patterns of progression. Previous studies have indicated that ALS commencing in a non-dominant limb tends to spread to the ipsilateral non-dominant limb, whereas that beginning in the dominant side spreads to the contralateral limb at that level.^42^

Compared with controls, the ALS group also showed predominant bilateral parietal atrophy. This finding has been suggested to indicate the presence of cases harbouring the *C9orf72* gene expansion, although recent studies caution that parietal atrophy is present in both *C9orf72* positive and negative cases.^46^ Further research is required to establish the nature of parietally driven symptoms in ALS, as previous research has tended to focus on frontal and temporal lobe functions. In terms of subcortical atrophy, volume loss was observed particularly in the bilateral pallidum, putamen, pons and brainstem.

Across the spectrum the most affected region in all three groups was the insula, and specifically the anterior part (76–90% lower than controls), potentially reflecting the emergence of physiological, autonomic and eating changes across these syndromes.^47^ The anterior insula is a major hub of the brain’s salience network with extensive links to both cortical and subcortical regions including the superior temporal pole, the dorsolateral prefrontal cortex, the amygdala, thalamus, hypothalamus and the substantia nigra/ventral tegmental area.^48^ Salience network dysfunction is a hallmark feature of bvFTD^49^ and has been proposed to underlie many of the core socioemotional and behavioural changes observed in this syndrome.^50^^,^^51^ The profound insular change observed in all groups in our study indicates the need for studies to explore the integrity of the other components of the salience network across the ALS–FTD spectrum.

By examining the neuroanatomical profiles across each clinical phenotype, we found evidence of common and unique cortical and subcortical signatures across the ALS–FTD spectrum. At the cortical level, both bvFTD and ALS–FTD were characterized by lower total frontal lobe volumes compared with ALS, while ALS was characterized by lower parietal lobe volumes, particularly the lateral parietal cortex compared with both ALS–FTD and bvFTD. ALS–FTD showed more temporal lobe atrophy compared with ALS, a finding which may underpin poorer language function in ALS–FTD compared with both ALS and bvFTD, which is often used as a clinical discriminator.^11^^,^^17^^,^^52^ The motor cortex was also a key differentiating structure, with bilateral motor cortex atrophy more pronounced in ALS–FTD compared with ALS, and left motor cortex atrophy greater in ALS–FTD compared with bvFTD. Previous studies have shown that ALS–FTD patients have greater atrophy of the motor cortex compared with pure ALS patients.^21^ Atrophy of the motor cortex is associated with a 1.5 times poorer survival across the ALS–FTD spectrum.^11^ Motor cortex dysfunction is also seen in bvFTD^20^ and thought to reflect hyperexcitability of the motor cortex shown on transcranial magnetic stimulation.^5^ In contrast, motor cortex atrophy is not consistently observed in pure ALS, with estimates of only 25% of patients showing frank atrophy.^44^^,^^53^ Given that both the ALS and the ALS–FTD groups had similar ALSFRS-R scores (a measure of functional decline, with a strong motor component), it seems unlikely that this change is due to greater motor involvement. As such, the clinical implication of greater motor cortex atrophy in ALS–FTD relative to the ALS group remains unclear. Further research is required to examine the relationship between motor cortex atrophy and disease progression, including motor function across the spectrum. The motor cortex has dense connections to many brain regions including the pyramidal corticospinal tracts, premotor cortex, parietal cortex, thalamus and cerebellum. The greater atrophy observed in ALS–FTD could be reflective of global network dysfunction, affecting overall physiological functioning^54^ and cognitive function, however this proposal will require further concerted investigation. Interestingly, increased left-sided insular atrophy was present in ALS–FTD compared with bvFTD, again potentially underpinning the importance of the insula cortices as a key structure in this syndrome.

At the subcortical level, ALS–FTD patients displayed more widespread subcortical involvement relative to ALS, with lower volumes of the putamen, caudate, amygdala and thalamus, likely explaining the presence of increased behavioural and neuropsychiatric symptoms in ALS–FTD compared with ALS.^55^ Likewise, bvFTD displayed smaller thalamus and right amygdala volumes relative to ALS, indicating that thalamic involvement may be a key marker of bvFTD that is not necessarily restricted to carriers of the *C9orf72* gene expansion, and may develop in ALS patients with frontal disease progression.^45^^,^^56^ ALS–FTD also showed smaller volumes in the caudate nucleus and the amygdala bilaterally compared with bvFTD, and this potentially explains the increased prevalence of emotion processing deficits seen in ALS–FTD compared with bvFTD.^11^ Traditionally, emotion processing and behavioural disturbances have been considered as core features of bvFTD and thought to be less prominent in ALS–FTD.^25^^,^^52^^,^^57^^,^^58^ The amygdala is key to emotion processing, motivation and reward and is consistently implicated in FTD.^59^^,^^60^ Further research is required to understand the nature of emotion processing and reward changes in ALS–FTD, as the suggestion is that these may be more prominent than first thought and not exclusively restricted to bvFTD. It is possible that the double hit of both ALS and FTD may in fact cause a more severe form of emotion dysregulation in ALS–FTD which could impact dramatically upon patients and their carers.

Our exploratory *C9orf72* positive versus negative analyses were largely commensurate with previous studies in FTD *C9orf72* expansion carriers, supporting distinct patterns with *C9orf72* FTD expansion carriers showing atrophy involving the frontotemporal, insular and parietal, occipital, thalamic and cerebellar regions, while individual cases can show more posterior atrophy.^61^^,^^62^ While exploratory analysis could not be conducted in the ALS group due to the small sample size, past studies suggest that *C9orf72* negative cases tend to show atrophy involving cortical frontal and temporal regions, while *C9orf72* ALS cases tend to show more subcortical involvement of the thalamus, caudate, putamen and pallidum.^63^ Future studies with a larger sample size of carriers of the *C9orf72* expansions will prove useful in validating the current findings and further differentiating *C9orf72* positive versus negative cases across the whole ALS–FTD spectrum.

Using the CBI-R, we next considered the multifaceted way in which behavioural symptoms coalesce across the ALS–FTD spectrum and their underlying neural correlates. Overall, behavioural change was greatest in bvFTD and ALS–FTD compared with ALS. In most domains, both ALS–FTD and bvFTD displayed disproportionate levels of behavioural changes compared with ALS. Key discriminating areas between ALS–FTD and bvFTD were eating changes, motivation and abnormal behaviours which were more prevalent in bvFTD compared with ALS–FTD. Across the ALS–FTD spectrum, abnormal behaviour including ritualistic behaviour, abnormal behaviour, hallucinations and increased eating behaviour correlated with smaller volumes in a frontoinsular, cingulate network previously implicated in such behaviours,^39^^,^^64^ extending to subcortical structures including the amygdala, ventral striatum and thalamus.

Finally, looking at cognitive profiles across the ALS–FTD spectrum, we found evidence of significant cognitive impairments across the majority of domains assessed in bvFTD and ALS–FTD patients relative to pure ALS, with disproportionate language impairments in ALS–FTD relative to bvFTD. Using a transdiagnostic approach, we found significant associations between all cognitive domains and volume loss in a distributed network of cortical and subcortical structures including frontoinsular, cingulate, insular and temporal regions and extending to the amygdala, ventral striatum and thalamus. These findings support the view that cognitive changes observed in neurodegenerative disorders are related to widespread network disintegration,^39^ rather than focal atrophy, which in turn has implications for our understanding of disease progression and the development of drug treatment targets. Further research is required to determine the precise unfolding of cognitive and behavioural symptoms in the face of progressive network degeneration, whether this process occurs in a stepwise fashion, and how this relates to disease staging and prognosis.

Key strengths of this study include our large sample size of well-characterized patients with standardized measures of cognition, behaviour and neuroimaging across the entire ALS–FTD spectrum. A number of methodological issues nevertheless warrant consideration. First, while we attempted to control for the potential confound of different scanners/imaging protocols used to obtain the images, there remains an imbalance in our group numbers, which may have influenced the study findings. In the absence of post mortem data, it is further possible that a proportion of bvFTD cases included in this study harboured tau and not TDP pathology, which may explain some of the volume differences observed. Currently in life there is no way of diagnosing underlying pathology (i.e. TDP-43 versus Tau pathology) in FTD. Once valid biomarkers are developed, we may be able to identify cases based on Tau versus TDP-43 pathology at the FTD end of the spectrum, which will prove useful in clinical imaging phenotyping. Future longitudinal studies should also examine the role that disease duration plays in the development of atrophy patterns and whether changes commence unilaterally and then progress bilaterally, as well as the pattern of spread of atrophy and how this relates to the development of cognitive and motor symptoms across the ALS–FTD spectrum. Future studies should also examine the relationship between brain atrophy and motor changes across the entire ALS–FTD spectrum taking disease severity into account. This is particularly challenging given the lack of verified measures that can be used to assess motor function and disease stage across the whole spectrum.

This study uncovers common and distinct atrophy profiles across the ALS–FTD spectrum and elucidates their relationship to the cognitive and behavioural disturbances observed in these syndromes. Our findings provide new insights into potential differentiators for improved diagnostic accuracy, such as the involvement of the motor cortex as a predictor of ALS–FTD, and disproportionate insula atrophy in ALS–FTD compared with bvFTD. Moreover, brainstem and pontine involvement may prove useful in differentiating ALS–FTD and ALS from bvFTD. Our finding of considerable insular cortex degeneration across the ALS–FTD spectrum suggests that functions relying on the insula may be deleteriously affected in these syndromes, which warrants careful consideration in future studies. Longitudinal studies will provide pivotal information regarding the fate of these atrophy profiles and their relationship to the emergence of motor, behavioural and cognitive symptoms with disease progression. These findings will help to elicit the true nature of the spectrum of ALS–FTD, and whether these conditions are separate entities with shared clinical, and atrophy profiles, or represent a true spectrum of disease progression. While we included a genetic component in the current study, investigation of pre-symptomatic genetic cohorts will be crucial to determine when different atrophy profiles develop and how they relate to the unfolding of distinct clinical symptoms. Ultimately, we propose that these efforts will promote a greater understanding of the diversity of cognitive, behavioural and imaging profiles across the ALS–FTD spectrum, enabling us to better identify and intervene to promote patient wellbeing and survival.

## Supplementary Material

fcab257_Supplementary_Data

## Abbreviations

ACE-III =: third edition of the Addenbrooke’s Cognitive Examination
ACE-R =: revised edition of the Addenbrooke’s Cognitive Examination
ALS =: amyotrophic lateral sclerosis
ALSFRS-R =: the revised ALS functional rating scale
ALS–FTD =: amyotrophic lateral sclerosis-frontotemporal dementia
bvFTD =: behavioural-variant frontotemporal dementia
CBI-R =: revised version of the Cambridge Behavioural Inventory
FAST =: FMRIB Automatic Segmentation Tool
FFE =: fast field echo
FRS =: Frontotemporal Dementia Rating Scale
FSL =: FMRIB Software Library
FTD =: frontotemporal dementia
FTD–ALS =: frontotemporal dementia–amyotrophic lateral sclerosis
GIF =: geodesic information flow
MNI152 =: Montreal Neurological Institute standard space
SPM12 =: Statistical Parametric Mapping 12
TMT =: trail making test
TIV =: total intracranial volume
