# Supplementary material for: Tackling clinical heterogeneity across the amyotrophic lateral sclerosis–frontotemporal dementia spectrum using a transdiagnostic approach
Source: Brain Commun. 2021 Oct 23;3(4):fcab257. doi: 10.1093/braincomms/fcab257 (PMC8599039; doi:10.1093/braincomms/fcab257)
Supplement: fcab257_Supplementary_Data [file fcab257_supplementary_data.docx]

| **Supplementary Table 1.** Brain volumes as percentage of TIV in bvFTD Patient Group compared to Controls | | | | |
| --- | --- | --- | --- | --- |
| **Brain Region** | **bvFTD**  **(*n* = 58)** | **Control**  **(*n* = 50)** | ***F*** | ***p*** |
| **Total Frontal Lobe** | 19.94(1.77) | 22.05(0.97) | 69.21 | <0.001 |
| Left | 9.94(0.96) | 10.70(0.47) | 42.146 | <0.001 |
| Right | 10.00(0.98) | 11.03(0.44) | 5.003 | <0.001 |
| **Dorsolateral Prefrontal Cortex** | |  |  |  |
| Left | 2.74(0.32) | 3.10(0.18) | 57.709 | <0.001 |
| Right | 2.73(0.34) | 3.07(0.17) | 47.994 | <0.001 |
| **Ventromedial Prefrontal Cortex** | |  |  |  |
| Left | 0.73(0.10) | 0.81(0.06) | 35.115 | <0.001 |
| Right | 0.86(0.12) | 0.99(0.08) | 48.459 | <0.001 |
| **Orbitofrontal Cortex** |  |  |  |  |
| Left | 0.61(0.09) | 0.70(0.06) | 37.652 | <0.001 |
| Right | 0.62(0.08) | 0.71(0.06) | 53.613 | <0.001 |
| **Motor Cortex** |  |  |  |  |
| Left | 1.28(0.11) | 1.36(0.09) | 17.627 | <0.001 |
| Right | 1.28(0.12) | 1.37(0.10) | 21.849 | <0.001 |
| **Total Cingulate Lobe** | 1.78(0.14) | 1.92(0.13) | 29.79 | <0.001 |
| Left | 0.93(0.07) | 1.00(0.07) | 3.854 | <0.001 |
| Right | 0.85(0.08) | 0.92(0.07) | 18.078 | <0.001 |
| **Anterior Cingulate** |  |  |  |  |
| Left | 0.61(0.07) | 0.67(0.06) | 25.254 | <0.001 |
| Right | 0.55(0.07) | 0.60(0.06) | 12.989 | <0.001 |
| **Middle Cingulate** |  |  |  |  |
| Left | 0.29(0.03) | 0.30(0.04) | 4.136 | 0.044 |
| Right | 0.30(0.03) | 0.32(0.04) | 1.075 | 0.002 |
| **Posterior Cingulate** |  |  |  |  |
| Left | 0.32(0.03) | 0.33(0.03) | 9.381 | 0.003 |
| Right | 0.30(0.03) | 0.32(0.03) | 11.696 | 0.001 |
| **Total Insular Lobe** | 0.59(0.11) | 0.70(0.06) | 54.635 | <0.001 |
| Left | 0.29(0.05) | 0.35(0.03) | 46.784 | <0.001 |
| Right | 0.30(0.06) | 0.35(0.03) | 52.187 | <0.001 |
| **Anterior Insula** |  |  |  |  |
| Left | 0.19(0.04) | 0.23(0.02) | 55.472 | <0.001 |
| Right | 0.19(0.04) | 0.24(0.02) | 53.726 | <0.001 |
| **Posterior Insula** |  |  |  |  |
| Left | 0.11(0.02) | 0.11(0.01) | 13.439 | <0.001 |
| Right | 0.11(0.02) | 0.12(0.01) | 24.119 | <0.001 |
| **Total Parietal Lobe** | 12.86(0.74) | 12.89(0.61) | 0.195 | 0.659 |
| Left | 6.46(0.37) | 6.43(0.33) | 0.010 | 0.992 |
| Right | 6.40(0.42) | 6.37(0.29) | 0.047 | 0.829 |
| **Medial Parietal Cortex** |  |  |  |  |
| Left | 0.64(0.06) | 0.65(0.06) | 1.07 | 0.303 |
| Right | 0.65(0.07) | 0.65(0.06) | 0.26 | 0.611 |
| **Lateral Parietal Cortex** |  |  |  |  |
| Left | 1.74(.18) | 1.78(.14) | 1.085 | 0.30 |
| Right | 1.69(.18) | 1.75(.13) | 4.124 | 0.045 |
| **Sensory Cortex** |  |  |  |  |
| Left | 0.70(0.08) | 0.70(0.07) | 0.003 | 0.954 |
| Right | 0.63(0.08) | 0.63(0.07) | 0.046 | 0.83 |
| **Total Temporal Lobe** | 12.34(0.96) | 13.06(0.57) | 23.428 | <0.001 |
| Left | 5.95(0.96) | 6.39(0.64) | 8.129 | 0.005 |
| Right | 6.01(0.98) | 6.51(0.62) | 1.23 | 0.002 |
| **Dorsolateral Temporal Cortex** | |  |  |  |
| Left | 2.49(.23) | 2.66(.15) | 23.484 | <0.001 |
| Right | 2.41(.25) | 2.64(.14) | 33.678 | <0.001 |
| **Medial Temporal Cortex** | |  |  |  |
| Left | 0.92(0.08) | 0.96(0.06) | 6.273 | 0.014 |
| Right | 0.93(0.08) | 0.97(0.06) | 12.536 | 0.001 |
| **Temporal Pole** |  |  |  |  |
| Left | 0.44(0.09) | 0.48(0.06) | 9.168 | 0.003 |
| Right | 0.42(0.08) | 0.48(0.04) | 22.954 | <0.001 |
| **Total Occipital Lobe** | 8.28(0.58) | 8.23(0.56) | 0.477 | 0.491 |
| Left | 4.05(0.31) | 4.06(0.29) | 0.206 | 0.651 |
| Right | 4.22(0.30) | 4.18(0.30) | 0.646 | 0.423 |
| **Hippocampus** |  |  |  |  |
| Left | 0.24(0.02) | 0.26(0.02) | 41.12 | <0.001 |
| Right | 0.24(0.03) | 0.27(0.02) | 39.143 | <0.001 |
| **Amygdala** |  |  |  |  |
| Left | 0.11(0.02) | 0.12(0.01) | 22.708 | <0.001 |
| Right | 0.10(0.02) | 0.12(0.01) | 26.977 | <0.001 |
| **Caudate** |  |  |  |  |
| Left | 0.20(0.03) | 0.20(0.02) | 0.787 | 0.377 |
| Right | 0.22(0.03) | 0.22(0.02) | 0.186 | 0.667 |
| **Putamen** |  |  |  |  |
| Left | 0.27(0.03) | 0.29(0.02) | 33.633 | <0.001 |
| Right | 0.27(0.03) | 0.29(0.02) | 18.247 | <0.001 |
| **Accumbens** |  |  |  |  |
| Left | 0.04(0.003) | 0.04(0.003) | 7.1 | 0.009 |
| Right | 0.04(0.003) | 0.04(0.003) | 6.106 | 0.015 |
| **Pallidum** |  |  |  |  |
| Left | 0.12(0.01) | 0.13(0.01) | 15.271 | <0.001 |
| Right | 0.12(0.01) | 0.13(0.01) | 12.724 | 0.001 |
| **Thalamus** |  |  |  |  |
| Left | 0.35(0.02) | 0.37(0.03) | 32.255 | <0.001 |
| Right | 0.34(0.02) | 0.36(0.03) | 32.438 | <0.001 |
| **Total Cerebellum** | 6.90(.50) | 7.10(.62) | 4.203 | 0.043 |
| Left | 3.13(.23) | 3.22(.29) | 4.875 | 0.029 |
| Right | 3.20(.24) | 3.29(.30) | 3.98 | 0.048 |
| **Vermis** | 0.32(0.03) | 0.32(0.03) | 0.043 | 0.836 |
| **Pons** | 0.68(0.08) | 0.68(0.06) | 0.496 | 0.483 |
| **Brainstem** | 0.56(0.04) | 0.55(0.04) | 0.06 | 0.807 |

*Values are Mean (Standard Deviation). bvFTD = behavioural variant Frontotemporal Dementia. TIV = Total Intracranial Volume.

| **Supplementary Table 2.** Brain volumes as percentage of TIV in ALS-FTD Patient Group compared to Controls | | | | |
| --- | --- | --- | --- | --- |
| **Brain Region** | **ALS-FTD**  **(*n* = 41)** | **Control**  **(*n* = 50)** | ***F*** | ***p*** |
| **Total Frontal Lobe** | 19.66(1.30) | 22.05(0.97) | 133.819 | <0.001 |
| Left | 9.77(0.64) | 10.70(0.47) | 87.566 | <0.001 |
| Right | 9.89(0.71) | 11.03(0.44) | 83.838 | <0.001 |
| **Dorsolateral Prefrontal Cortex** | |  |  |  |
| Left | 2.72(0.17) | 3.10(0.18) | 92.516 | <0.001 |
| Right | 2.74(0.20) | 3.07(0.17) | 78.263 | <0.001 |
| **Ventromedial Prefrontal Cortex** | |  |  |  |
| Left | 0.72(0.07) | 0.81(0.06) | 36.86 | <0.001 |
| Right | 0.87(0.10) | 0.99(0.08) | 47.295 | <0.001 |
| **Orbitofrontal Cortex** |  |  |  |  |
| Left | 0.59(0.06) | 0.70(0.06) | 7.817 | <0.001 |
| Right | 0.62(0.06) | 0.71(0.06) | 53.37 | <0.001 |
| **Motor Cortex** |  |  |  |  |
| Left | 1.23(0.10) | 1.36(0.09) | 36.495 | <0.001 |
| Right | 1.22(0.10) | 1.37(0.10) | 45.617 | <0.001 |
| **Total Cingulate Lobe** | 1.82(0.12) | 1.92(0.13) | 11.305 | 0.001 |
| Left | 0.94(0.06) | 1.00(0.07) | 16.536 | <0.001 |
| Right | 0.88(0.07) | 0.92(0.07) | 3.714 | 0.057 |
| **Anterior Cingulate** |  |  |  |  |
| Left | 0.61(0.05) | 0.67(0.06) | 2.867 | <0.001 |
| Right | 0.56(0.06) | 0.60(0.06) | 4.514 | 0.036 |
| **Middle Cingulate** |  |  |  |  |
| Left | 0.29(0.03) | 0.30(0.04) | 1.394 | 0.241 |
| Right | 0.31(0.04) | 0.32(0.04) | 2.007 | 0.16 |
| **Posterior Cingulate** |  |  |  |  |
| Left | 0.32(0.03) | 0.33(0.03) | 1.263 | 0.264 |
| Right | 0.32(0.03) | 0.32(0.03) | 0.072 | 0.789 |
| **Total Insular Lobe** | 0.56(0.08) | 0.70(0.06) | 128.813 | <0.001 |
| Left | 0.27(0.04) | 0.35(0.03) | 118.038 | <0.001 |
| Right | 0.29(0.04) | 0.35(0.03) | 101.723 | <0.001 |
| **Anterior Insula** |  |  |  |  |
| Left | 0.18(0.03) | 0.23(0.02) | 128.513 | <0.001 |
| Right | 0.19(0.03) | 0.24(0.02) | 9.454 | <0.001 |
| **Posterior Insula** |  |  |  |  |
| Left | 0.10(0.02) | 0.11(0.01) | 4.086 | <0.001 |
| Right | 0.10(0.02) | 0.12(0.01) | 43.087 | <0.001 |
| **Total Parietal Lobe** | 12.88(.94) | 12.89(.61) | 0.01 | 0.923 |
| Left | 6.49(.50) | 6.43(.33) | 0.177 | 0.675 |
| Right | 6.39(.46) | 6.37(.29) | 0.003 | 0.953 |
| **Medial Parietal Cortex** |  |  |  |  |
| Left | 0.67(0.08) | 0.65(0.06) | 2.143 | 0.147 |
| Right | 0.66(0.06) | 0.65(0.06) | 0.328 | 0.568 |
| **Lateral Parietal Cortex** |  |  |  |  |
| Left | 1.75(.18) | 1.78(.14) | 0.023 | 0.88 |
| Right | 1.70(.17) | 1.75(.13) | 2.094 | 0.151 |
| **Sensory Cortex** |  |  |  |  |
| Left | 0.70(0.07) | 0.70(0.07) | 0.09 | 0.765 |
| Right | 0.65(0.08) | 0.63(0.07) | 1.553 | 0.216 |
| **Total Temporal Lobe** | 12.25(1.10) | 13.06(.57) | 25.883 | <0.001 |
| Left | 5.66(1.22) | 6.39(.64) | 15.461 | <0.001 |
| Right | 5.86(1.30) | 6.51(.62) | 11.341 | 0.001 |
| **Dorsolateral Temporal Cortex** | |  |  |  |
| Left | 2.41(.24) | 2.66(.15) | 35.902 | <0.001 |
| Right | 2.42(.25) | 2.64(.14) | 27.468 | <0.001 |
| **Medial Temporal Cortex** | |  |  |  |
| Left | 0.89(0.10) | 0.96(0.06) | 16.981 | <0.001 |
| Right | 0.92(0.11) | 0.97(0.06) | 16.157 | <0.001 |
| **Temporal Pole** |  |  |  |  |
| Left | 0.41(0.09) | 0.48(0.06) | 25.783 | <0.001 |
| Right | 0.42(0.10) | 0.48(0.04) | 27.098 | <0.001 |
| **Total Occipital Lobe** | 8.34(.61) | 8.23(.56) | 0.408 | 0.525 |
| Left | 3.99(.49) | 4.06(.29) | 0.151 | 0.699 |
| Right | 4.13(.51) | 4.18(.30) | 0.257 | 0.613 |
| **Hippocampus** |  |  |  |  |
| Left | 0.23(0.03) | 0.26(0.02) | 6.785 | <0.001 |
| Right | 0.24(0.03) | 0.27(0.02) | 35.504 | <0.001 |
| **Amygdala** |  |  |  |  |
| Left | 0.10(0.02) | 0.12(0.01) | 47.609 | <0.001 |
| Right | 0.10(0.01) | 0.12(0.01) | 59.273 | <0.001 |
| **Caudate** |  |  |  |  |
| Left | 0.19(0.03) | 0.20(0.02) | 13.732 | <0.001 |
| Right | 0.20(0.03) | 0.22(0.02) | 12.748 | 0.001 |
| **Putamen** |  |  |  |  |
| Left | 0.26(0.02) | 0.29(0.02) | 56.885 | <0.001 |
| Right | 0.26(0.02) | 0.29(0.02) | 29.462 | <0.001 |
| **Accumbens** |  |  |  |  |
| Left | 0.04(0.004) | 0.04(0.003) | 12.358 | 0.001 |
| Right | 0.03(0.004) | 0.04(0.003) | 9.372 | 0.003 |
| **Pallidum** |  |  |  |  |
| Left | 0.12(0.01) | 0.13(0.01) | 19.782 | <0.001 |
| Right | 0.12(0.01) | 0.13(0.01) | 12.196 | 0.001 |
| **Thalamus** |  |  |  |  |
| Left | 0.34(0.03) | 0.37(0.03) | 25.679 | <0.001 |
| Right | 0.34(0.03) | 0.36(0.03) | 2.725 | <0.001 |
| **Total Cerebellum** | 6.76(.54) | 7.10(.62) | 6.751 | 0.011 |
| Left | 3.08(.25) | 3.22(.29) | 5.96 | 0.017 |
| Right | 3.12(.26) | 3.29(.30) | 7.343 | 0.008 |
| **Vermis** | 0.32(0.03) | 0.32(0.03) | 0.571 | 0.452 |
| **Pons** | 0.64(0.07) | 0.68(0.06) | 12.669 | 0.001 |
| **Brainstem** | 0.54(0.04) | 0.55(0.04) | 5.035 | 0.027 |

*Values are Mean (Standard Deviation). ALS-FTD = Amyotrophic Lateral sclerosis- Frontotemporal Dementia. TIV = Total Intracranial Volume.

| **Supplementary Table 3.** Brain volumes as percentage of TIV in ALS Patient Group compared to Controls | | |  |  |
| --- | --- | --- | --- | --- |
| **Brain Region** | **ALS**  **(*n* = 52)** | **Control**  **(*n* = 8)** | ***F*** | ***p*** |
| **Total Frontal Lobe** | 21.88(1.38) | 22.96(1.09) | 3.252 | 0.074 |
| Left | 10.91(0.68) | 11.47(0.56) | 0.572 | 0.453 |
| Right | 10.97(0.72) | 11.48(0.54) | 0.366 | 0.548 |
| **Dorsolateral Prefrontal Cortex** | |  |  |  |
| Left | 2.94(0.23) | 3.25(0.23) | 14.62 | <0.001 |
| Right | 2.92(0.22) | 3.16(0.21) | 16.391 | <0.001 |
| **Ventromedial Prefrontal Cortex** | |  |  |  |
| Left | 0.74(0.07) | 0.83(0.07) | 26.961 | <0.001 |
| Right | 0.90(0.07) | 1.02(0.07) | 28.231 | <0.001 |
| **Orbitofrontal Cortex** | |  |  |  |
| Left | 0.68(0.05) | 0.76(0.09) | 1.981 | 0.162 |
| Right | 0.69(0.06) | 0.76(0.07) | 1.882 | 0.173 |
| **Motor Cortex** |  |  |  |  |
| Left | 1.31(0.12) | 1.36(0.08) | 4.718 | 0.032 |
| Right | 1.31(0.16) | 1.36(0.08) | 8.48 | 0.004 |
| **Total Cingulate Lobe** | 1.97(0.13) | 1.97(0.12) | 4.583 | 0.035 |
| Left | 1.02(0.08) | 1.05(0.07) | 1.688 | 0.197 |
| Right | 0.94(0.07) | 0.96(0.06) | 6.807 | 0.01 |
| **Anterior Cingulate** |  |  |  |  |
| Left | 0.68(0.06) | 0.70(0.06) | 1.088 | 0.299 |
| Right | 0.61(0.06) | 0.60(0.05) | 3.095 | 0.081 |
| **Middle Cingulate** |  |  |  |  |
| Left | 0.31(0.05) | 0.32(0.04) | 2.355 | 0.128 |
| Right | 0.33(0.04) | 0.34(0.04) | 4.144 | 0.044 |
| **Posterior Cingulate** |  |  |  |  |
| Left | 0.34(0.03) | 0.35(0.02) | 1.382 | 0.242 |
| Right | 0.34(0.03) | 0.36(0.02) | 7.154 | 0.009 |
| **Total Insular Lobe** | 0.69(0.07) | 0.76(0.05) | 3.348 | 0.07 |
| Left | 0.34(0.03) | 0.38(0.02) | 0.815 | 0.369 |
| Right | 0.35(0.04) | 0.38(0.02) | 6.189 | 0.014 |
| **Anterior Insula** |  |  |  |  |
| Left | 0.23(0.02) | 0.25(0.02) | 2.654 | 0.106 |
| Right | 0.23(0.03) | 0.26(0.02) | 5.122 | 0.026 |
| **Posterior Insula** |  |  |  |  |
| Left | 0.12(0.01) | 0.13(0.01) | 0.717 | 0.399 |
| Right | 0.12(0.02) | 0.13(0.01) | 3.761 | 0.055 |
| **Total Parietal Lobe** | 13.36(0.76) | 13.49(0.32) | 8.797 | 0.004 |
| Left | 6.71(0.36) | 6.79(0.15) | 0.557 | 0.549 |
| Right | 6.65(0.42) | 6.70(0.19) | 0.874 | 0.354 |
| **Medial Parietal Cortex** | |  |  |  |
| Left | 0.65(0.06) | 0.68(0.05) | 0.377 | 0.54 |
| Right | 0.67(0.06) | 0.66(0.06) | 0.075 | 0.785 |
| **Lateral Parietal Cortex** | |  |  |  |
| Left | 1.63(0.20) | 1.8(0.19) | 15.012 | <0.001 |
| Right | 1.60(0.18) | 1.78(0.17) | 19.879 | <0.001 |
| **Sensory Cortex** |  |  |  |  |
| Left | 0.64(0.08) | 0.69(0.06) | 1.33 | 0.002 |
| Right | 0.57(0.08) | 0.63(0.06) | 11.799 | 0.001 |
| **Total Temporal Lobe** | 13.29(0.77) | 13.64(0.40) | 0.352 | 0.556 |
| Left | 6.61(0.40) | 6.73(0.22) | 0.002 | 0.966 |
| Right | 6.68(0.39) | 6.92(0.19) | 0.065 | 0.800 |
| **Dorsolateral Temporal Cortex** | |  |  |  |
| Left | 2.60(0.16) | 2.74(0.15) | 0.86 | 0.359 |
| Right | 2.53(0.25) | 2.73(0.14) | 0.179 | 0.674 |
| **Medial Temporal Cortex** | |  |  |  |
| Left | 1.02(0.06) | 1.02(0.05) | 0.362 | 0.551 |
| Right | 1.01(0.06) | 1.06(0.06) | 3.325 | 0.075 |
| **Temporal Pole** |  |  |  |  |
| Left | 0.49(0.07) | 0.53(0.08) | 1.23 | 0.274 |
| Right | 0.48(0.07) | 0.54(0.08) | 2.736 | 0.106 |
| **Total Occipital Lobe** | 8.49(0.58) | 8.73(0.36) | 0.003 | 0.959 |
| Left | 3.72(0.76) | 4.03(0.60) | 1.603 | 0.211 |
| Right | 3.96(0.83) | 4.27(0.70) | 1.470 | 0.231 |
| **Hippocampus** |  |  |  |  |
| Left | 0.26(0.02) | 0.26(0.02) | 0.186 | 0.668 |
| Right | 0.27(0.02) | 0.27(0.02) | 0.736 | 0.395 |
| **Amygdala** |  |  |  |  |
| Left | 0.12(0.01) | 0.13(0.01) | 3.741 | 0.056 |
| Right | 0.12(0.01) | 0.12(0.01) | 0.001 | 0.982 |
| **Caudate** |  |  |  |  |
| Left | 0.21(0.02) | 0.22(0.01) | 1.042 | 0.313 |
| Right | 0.23(0.03) | 0.23(0.02) | 0.349 | 0.558 |
| **Putamen** |  |  |  |  |
| Left | 0.28(0.02) | 0.29(0.02) | 1.094 | 0.002 |
| Right | 0.28(0.02) | 0.29(0.02) | 8.082 | 0.005 |
| **Accumbens** |  |  |  |  |
| Left | 0.04(0.002) | 0.04(0.003) | 3.413 | 0.068 |
| Right | 0.04(0.002) | 0.04(0.003) | 1.024 | 0.314 |
| **Pallidum** |  |  |  |  |
| Left | 0.12(0.01) | 0.13(0.01) | 24.936 | <0.001 |
| Right | 0.12(0.01) | 0.13(0.01) | 18.157 | <0.001 |
| **Thalamus** |  |  |  |  |
| Left | 0.38(0.03) | 0.39(0.04) | 0.966 | 0.328 |
| Right | 0.37(0.03) | 0.38(0.04) | 0.663 | 0.417 |
| **Total Cerebellum** | 6.93(0.56) | 7.0(0.89) | 2.097 | 0.151 |
| Left | 3.13(0.26) | 3.16(0.42) | 3.48 | 0.065 |
| Right | 3.23(0.26) | 3.26(0.41) | 1.33 | 0.251 |
| **Vermis** | 0.31(0.03) | 0.31(0.05) | 0.047 | 0.828 |
| **Pons** | 0.64(0.05) | 0.67(0.1) | 22.007 | <0.001 |
| **Brainstem** | 0.52(0.03) | 0.53(0.06) | 29.654 | <0.001 |

*Values are Mean (Standard Deviation). ALS = Amyotrophic Lateral Sclerosis. TIV = Total Intracranial Volume.

| **Supplementary Table 4.** Brain volume differences in bvFTD patients with and without *C9orf72* expansion | | | | | |  |
| --- | --- | --- | --- | --- | --- | --- |
| **Brain Region** | **bvFTD with c9orf72**  **(*n* = 17)** | **bvFTD without c9orf72**  **(*n* = 41)** | | ***t*** | ***p*** |  |
| **Total Frontal Lobe** | 281,377.46(34,847.50) | 294,946.40(35,691.91) | 1.178 | | 0.244 |  |
| Left | 140,080.45(15,887.92) | 147,036.04(18,910.17) | 1.169 | | 0.247 |  |
| Right | 141,297.02(19,425.68) | 147,910.36(18,879.08) | 1.074 | | 0.287 |  |
| **Dorsolateral Prefrontal Cortex** | |  |  | |  |  |
| Left | 39,099.99(5,853.65) | 40,371.62(5,126.61) | 0.743 | | 0.46 |  |
| Right | 39,388.24(6,335.30) | 40,064.71(5,767.18) | 0.355 | | 0.724 |  |
| **Ventromedial Prefrontal Cortex** | |  |  | |  |  |
| Left | 10,696.99(1,535.84) | 10,666.93 (1,461.36) | -0.063 | | 0.95 |  |
| Right | 12,671.12(1,736.30) | 12,548.11(1,983.42) | -0.196 | | 0.845 |  |
| **Orbitofrontal Cortex** |  |  |  | |  |  |
| Left | 8,750.48(1,159.31) | 9,050.80(1,510.14) | 0.64 | | 0.525 |  |
| Right | 8,853.62(1,144.96) | 9,121.73(1,341.19) | 0.634 | | 0.529 |  |
| **Motor Cortex** |  |  |  | |  |  |
| Left | 17,723.56(2,413.66) | 18,934.02(2,095.59) | 1.727 | | 0.09 |  |
| Right | 17,956.60(2,726.19) | 18,911.08(2,163.02) | 1.289 | | 0.203 |  |
| **Total Cingulate Lobe** | 26,056.55(2,692.06) | 26,122.83(3,013.46) | 0.069 | | 0.945 |  |
| Left | 13,557.26(1,501.35) | 13,643.62(1,564.11) | 0.172 | | 0.864 |  |
| Right | 12,499.29(1,281.16) | 12,479.21(1,650.93) | -0.039 | | 0.969 |  |
| **Anterior Cingulate** |  |  |  | |  |  |
| Left | 9,009.74(1,046.85) | 8,970.95(1,190.05) | -0.103 | | 0.918 |  |
| Right | 8,163.03(762.26) | 8,025.08(1,220.61) | -0.372 | | 0.712 |  |
| **Middle Cingulate** |  |  |  | |  |  |
| Left | 4,263.87(414.34) | 4,221.38(487.62) | -0.276 | | 0.783 |  |
| Right | 4,379.61(333.61) | 4,414.54(586.79) | 0.197 | | 0.844 |  |
| **Posterior Cingulate** |  |  |  | |  |  |
| Left | 4,547.52(588.01) | 4,672.67(633.30) | 0.618 | | 0.539 |  |
| Right | 4,336.26(592.22) | 4,454.14(591.70) | 0.614 | | 0.541 |  |
| **Total Insular Lobe** | 8,519.04(1,088.43) | 8,724.57(1,904.78) | 0.488^a^ | | 0.629 |  |
| Left | 4,168.82(638.73) | 4,330.95(942.81) | 0.561 | | 0.577 |  |
| Right | 4,350.22(501.80) | 4,393.63(1,021.52) | 0.208^a^ | | 0.837 |  |
| **Anterior Insula** |  |  |  | |  |  |
| Left | 2,764.96(487.65) | 2,755.24(704.80) | -0.045 | | 0.964 |  |
| Right | 2,847.87(382.64) | 2,809.58(760.93) | -0.243^a^ | | 0.809 |  |
| **Posterior Insula** |  |  |  | |  |  |
| Left | 1,403.85(192.28) | 1,575.71(276.20) | 2.025 | | 0.048 |  |
| Right | 1,502.35(157.61) | 1,584.05(304.57) | 1.278^a^ | | 0.21 |  |
| **Total Parietal Lobe** | 179,134.84(16,049.30) | 190,905.53(19,276.50) | 1.943 | | 0.057 |  |
| Left | 90,686.60(7,942.00) | 95,746.39(9,620.71) | 1.676 | | 0.099 |  |
| Right | 88,448.24(8,307.64) | 95,159.14(10,160.98) | 2.107 | | 0.04 |  |
| **Medial Parietal Cortex** | |  |  | |  |  |
| Left | 9,253.17(1,489.11) | 9,484.06(1,043.38) | 0.622 | | 0.536 |  |
| Right | 9,031.07(1,348.38) | 9,629.24(1,132.04) | 1.567 | | 0.123 |  |
| **Lateral Parietal Cortex** | |  |  | |  |  |
| Left | 23,898.41(2,669.26) | 25,802.95(2,830.28) | 2.099 | | 0.04 |  |
| Right | 22,998.39(2,361.72) | 25,126.71(3,082.01) | 2.222 | | 0.03 |  |
| **Sensory Cortex** |  |  |  | |  |  |
| Left | 9,762.85 (1,479.03) | 10,276.11 (1,184.70) | 1.269 | | 0.21 |  |
| Right | 8,853.26(1,009.51) | 9,205.13(1,227.45) | 0.914 | | 0.365 |  |
| **Total Temporal Lobe** | 172,041.80(11,385.90) | 181,945.36(21,939.77) | 2.077^a^ | | 0.046 |  |
| Left | 81,340.93(15,217.48) | 88,471.96(15,040.19) | 1.459 | | 0.15 |  |
| Right | 82,642.27(14,857.89) | 89,122.10(15,609.51) | 1.293 | | 0.201 |  |
| **Dorsolateral Temporal Cortex** | |  |  | |  |  |
| Left | 34,434.11(2,861.85) | 36,659.64(4,214.90) | 2.077^a^ | | 0.05 |  |
| Right | 33,981.46(2,531.38) | 35,511.26(5,053.06) | 1.419^a^ | | 0.166 |  |
| **Medial Temporal Cortex** | |  |  | |  |  |
| Left | 12,567.90(913.58) | 13,701.22(1,904.11) | 2.849^a^ | | 0.007 |  |
| Right | 12,891.80(924.66) | 13,750.44(1,956.75) | 2.115^a^ | | 0.042 |  |
| **Temporal Pole** |  |  |  | |  |  |
| Left | 6,077.66(1,144.87) | 6,473.16(1,477.64) | 0.826 | | 0.413 |  |
| Right | 5,882.31(974.72) | 6,185.21(1,489.05) | 0.639 | | 0.526 |  |
| **Total Occipital Lobe** | 117,123.96(10,171.37) | 122,342.73(13,871.90) | 1.217 | | 0.229 |  |
| Left | 57,459.18(5,322.86) | 59,870.86(6,962.83) | 1.115 | | 0.27 |  |
| Right | 59,664.78(5,089.22) | 62,471.87(7,230.11) | 1.262 | | 0.212 |  |
| **Hippocampus** |  |  |  | |  |  |
| Left | 3,433.02(339.19) | 3,519.47(445.79) | 0.625 | | 0.535 |  |
| Right | 3,551.43(339.07) | 3,562.11(520.30) | 0.067 | | 0.947 |  |
| **Amygdala** |  |  |  | |  |  |
| Left | 1,456.76(210.29) | 1,584.36(307.82) | 1.352 | | 0.182 |  |
| Right | 1,452.10(171.54) | 1,551.07(292.88) | 1.117 | | 0.269 |  |
| **Caudate** |  |  |  | |  |  |
| Left | 2,858.18(420.14) | 2,947.84(501.80) | 0.568 | | 0.572 |  |
| Right | 3,043.91(481.65) | 3,209.32(571.76) | 0.919 | | 0.362 |  |
| **Putamen** |  |  |  | |  |  |
| Left | 3,794.84(452.63) | 3,956.27(471.30) | 1.065 | | 0.292 |  |
| Right | 3,764.91(534.12) | 3,938.24(523.03) | 1.018 | | 0.313 |  |
| **Accumbens** |  |  |  | |  |  |
| Left | 521.06(43.90) | 540.46(69.28) | 1.192^a^ | | 0.244 |  |
| Right | 516.06(45.41) | 518.35(68.46) | 0.11 | | 0.913 |  |
| **Pallidum** |  |  |  | |  |  |
| Left | 1,750.18(222.89) | 1,811.66(187.42) | 0.973 | | 0.335 |  |
| Right | 1,743.17(219.59) | 1,831.45(211.55) | 1.278 | | 0.207 |  |
| **Thalamus** |  |  |  | |  |  |
| Left | 4,839.12(437.20) | 5,173.75(520.12) | 2.045 | | 0.046 |  |
| Right | 4,733.19(418.01) | 5,033.68(521.08) | 1.845 | | 0.07 |  |
| **Total Cerebellum** | 97,486.09(10,770.36) | 101,938.88(9,500.88) | 1.407 | | 0.165 |  |
| Left | 44,046.00(5,059.36) | 46,226.50(4,488.40) | 1.46 | | 0.15 |  |
| Right | 45,112.12(5,173.04) | 47,276.13(4,632.09) | 1.407 | | 0.165 |  |
| **Vermis** | 4,557.72(397.47) | 4,657.28(471.87) | 0.67 | | 0.505 |  |
| **Pons** | 10,091.13(1,326.42) | 9,863.71(1,241.35) | -0.557 | | 0.579 |  |
| **Brainstem** | 8,269.03(884.80) | 8,089.91(819.62) | -0.664 | | 0.51 |  |

*Values are Mean (Standard Deviation). ^a^Independent sample *t*-test value (equal variances not assumed)

bvFTD = behavioural variant Frontotemporal Dementia.

| **Supplementary Table 5.** Brain volume differences in ALS-FTD patients with and without *C9orf72* expansion | | | | |  |
| --- | --- | --- | --- | --- | --- |
| **Brain Region** | **ALS-FTD with *C9or72***  **(*n* = 12)** | **ALS-FTD without *C9orf72***  **(*n* = 29)** | ***t*** | ***p*** |  |
| **Total Frontal Lobe** | 285,408.87(37,245.96) | 299,585.89(41,062.25) | 1.032 | 0.308 |  |
| Left | 142,638.63(19,632.30) | 148,633.77(19,972.61) | 0.879 | 0.385 |  |
| Right | 142,770.24(18,090.76) | 150,952.12(21,487.75) | 1.158 | 0.254 |  |
| **Dorsolateral Prefrontal Cortex** | |  |  |  |  |
| Left | 40,440.07(5,604.54) | 41,008.69(5,119.75) | 0.315 | 0.755 |  |
| Right | 40,377.00(4,956.37) | 41,412.58(6,091.48) | 0.521 | 0.605 |  |
| **Ventromedial Prefrontal Cortex** | |  |  |  |  |
| Left | 10232.92(1,482.35) | 11115.53(1,670.44) | 1.588 | 0.12 |  |
| Right | 12,301.71(1,685.13) | 13,294.95(2,229.13) | 1.384 | 0.174 |  |
| **Orbitofrontal Cortex** |  |  |  |  |  |
| Left | 8,342.62(899.54) | 9,031.46(1,186.72) | 1.803 | 0.079 |  |
| Right | 8,918.81(1,038.94) | 9,328.29(1,162.32) | 1.057 | 0.297 |  |
| **Motor Cortex** |  |  |  |  |  |
| Left | 17,787.07(1,837.47) | 19,360.06(3,288.96) | 1.945^a^ | 0.06 |  |
| Right | 18,144.99(1,903.62) | 19,059.74(3,019.91) | 0.969 | 0.339 |  |
| **Total Cingulate Lobe** | 26,395.71(3,940.06) | 27,589.20(3,149.05) | 1.025 | 0.311 |  |
| Left | 13,707.99(1,916.63) | 14,205.61(1,890.78) | 0.764 | 0.45 |  |
| Right | 12,687.71(2,081.70) | 13,383.59(1,464.76) | 1.22 | 0.23 |  |
| **Anterior Cingulate** |  |  |  |  |  |
| Left | 8,946.11(1,129.83) | 9,282.33(1,343.74) | 0.761 | 0.451 |  |
| Right | 8,088.83(1,310.38) | 8,578.36(1,035.20) | 1.274 | 0.21 |  |
| **Middle Cingulate** |  |  |  |  |  |
| Left | 4,322.86(627.99) | 4,358.36(677.09) | 0.156 | 0.877 |  |
| Right | 4,527.57(773.39) | 4,692.74(514.99) | 0.803 | 0.427 |  |
| **Posterior Cingulate** |  |  |  |  |  |
| Left | 4,761.89(834.26) | 4,923.28(646.83) | 0.667 | 0.509 |  |
| Right | 4,598.89(901.09) | 4,805.23(615.57) | 0.849 | 0.401 |  |
| **Total Insular Lobe** | 8,201.13(824.06) | 8,533.37(1,509.53) | 0.904^a^ | 0.372 |  |
| Left | 3,940.22(497.58) | 4,135.98(712.12) | 0.866 | 0.392 |  |
| Right | 4,260.90(458.58) | 4,397.39(849.00) | 0.524 | 0.604 |  |
| **Anterior Insula** |  |  |  |  |  |
| Left | 2,583.35(362.56) | 2,666.94(482.35) | 0.539 | 0.593 |  |
| Right | 2,803.24(331.84) | 2,839.22(581.60) | 0.249^a^ | 0.805 |  |
| **Posterior Insula** |  |  |  |  |  |
| Left | 1,356.87(217.79) | 1,469.04(279.04) | 1.242 | 0.222 |  |
| Right | 1,457.67(232.94) | 1,558.18(319.67) | 0.983 | 0.331 |  |
| **Total Parietal Lobe** | 186,915.24(29,773.30) | 196,177.00(25,338.38) | 1.012 | 0.318 |  |
| Left | 94,749.06(16,292.06) | 98,675.91(13,036.76) | 0.815 | 0.42 |  |
| Right | 92,166.18(13,524.01) | 97,501.09(12,553.00) | 1.211 | 0.233 |  |
| **Medial Parietal Cortex** | |  |  |  |  |
| Left | 9,977.82(2,355.19) | 10,181.17(1,371.00) | 0.28^a^ | 0.783 |  |
| Right | 9,627.19(1,686.82) | 10,043.27(1,387.66) | 0.82 | 0.417 |  |
| **Lateral Parietal Cortex** | |  |  |  |  |
| Left | 24,918.85(2,945.76) | 26,692.03(3,947.81) | 1.399 | 0.17 |  |
| Right | 24,334.88(2,626.75) | 26,005.01(3,916.43) | 1.352 | 0.184 |  |
| **Sensory Cortex** |  |  |  |  |  |
| Left | 10099.08(1,100.06) | 10726.17(1,451.21) | 1.342 | 0.187 |  |
| Right | 9348.57 (1,020.03) | 9775.60(1,467.32) | 0.917 | 0.365 |  |
| **Total Temporal Lobe** | 179,002.25(28,162.24) | 185,311.89(25,970.93) | 0.673 | 0.505 |  |
| Left | 89,570.96(14,032.83) | 82,751.67(22,400.86) | -0.974 | 0.336 |  |
| Right | 89,431.29(14630.43) | 87,147.73(24079.93) | -0.305 | 0.762 |  |
| **Dorsolateral Temporal Cortex** | |  |  |  |  |
| Left | 35,488.83(5,004.04) | 36,313.63(5,288.77) | 0.452 | 0.654 |  |
| Right | 34,514.05(4,789.25) | 36,853.90(5,471.76) | 1.265 | 0.214 |  |
| **Medial Temporal Cortex** | |  |  |  |  |
| Left | 13,079.96(2,205.19) | 13,427.28(2,220.72) | 0.446 | 0.658 |  |
| Right | 13,343.87(2,337.95) | 13,890.17(2,255.12) | 0.682 | 0.5 |  |
| **Temporal Pole** |  |  |  |  |  |
| Left | 6,328.76(1,329.86) | 5,941.05(1,563.88) | -0.739 | 0.465 |  |
| Right | 6,376.81(1,854.59) | 6,258.36(1,552.78) | -0.204 | 0.84 |  |
| **Total Occipital Lobe** | 115,687.63(14,511.44) | 127,588.26(14,294.45) | 2.251 | 0.031 |  |
| Left | 54,995.83(7,778.76) | 61,653.24(8,561.17) | 2.323 | 0.025 |  |
| Right | 56,852.86(8,253.16) | 63,983.95(9,115.25) | 2.339 | 0.025 |  |
| **Hippocampus** |  |  |  |  |  |
| Left | 3,177.42(439.79) | 3,521.03(475.18) | 2.151 | 0.038 |  |
| Right | 3,241.71(357.42) | 3,764.45(519.60) | 3.176 | 0.003 |  |
| **Amygdala** |  |  |  |  |  |
| Left | 1,424.79(193.25) | 1,474.88(306.80) | 0.522 | 0.605 |  |
| Right | 1,388.79(224.58) | 1,482.33(248.58) | 1.126 | 0.267 |  |
| **Caudate** |  |  |  |  |  |
| Left | 2,659.74(484.28) | 2,898.73(617.54) | 1.194 | 0.24 |  |
| Right | 2,883.29(534.89) | 3,173.74(712.70) | 1.268 | 0.212 |  |
| **Putamen** |  |  |  |  |  |
| Left | 3,651.42(426.62) | 4,018.50(397.55) | 2.634 | 0.012 |  |
| Right | 3,652.13(380.82) | 4,057.51(432.02) | 2.824 | 0.007 |  |
| **Accumbens** |  |  |  |  |  |
| Left | 499.97(65.78) | 548.79(66.30) | 2.15 | 0.038 |  |
| Right | 480.97(66.92) | 538.89(72.65) | 2.374 | 0.023 |  |
| **Pallidum** |  |  |  |  |  |
| Left | 1,698.71(180.70) | 1,863.50(165.08) | 2.83 | 0.007 |  |
| Right | 1,684.04(184.63) | 1,905.28(178.85) | 3.571 | 0.001 |  |
| **Thalamus** |  |  |  |  |  |
| Left | 4,711.50(659.53) | 5,270.13(501.54) | 2.955 | 0.005 |  |
| Right | 4,635.71(632.65) | 5,175.61(501.25) | 2.905 | 0.006 |  |
| **Total Cerebellum** | 97,356.50(10,431.81) | 102,733.83(10,258.72) | 1.52 | 0.137 |  |
| Left | 44,387.26(4,980.49) | 46,787.24(4,833.36) | 1.434 | 0.159 |  |
| Right | 44,970.66(4,751.18) | 47,453.44(4,828.83) | 1.505 | 0.14 |  |
| **Vermis** | 4,452.69(426.54) | 4,856.44(422.62) | 2.776 | 0.008 |  |
| **Pons** | 9,468.80(1,389.63) | 9,601.71(1,375.61) | 0.281 | 0.78 |  |
| **Brainstem** | 8,031.20(888.69) | 8,039.03(844.14) | 0.027 | 0.979 |  |

*Values are Mean (Standard Deviation). ^a^Independent sample *t*-test value (equal variances not assumed)

ALS-FTD = amyotrophic lateral sclerosis-frontotemporal Dementia.

**Supplementary Table 6.** Pearson’s Correlations between Brain Region Volumes and Cognitive Variables across all patient groups

|  | **ACE Attention** | | **ACE Memory** | **ACE Fluency** | | **ACE Language** | | **ACE Visuospatial** | | **ACE Total** | | **TMT-A** | | **TMT-**  **B-A** |
| --- | --- | --- | --- | --- | --- | --- | --- | --- | --- | --- | --- | --- | --- | --- |
| **Total Frontal Lobe** | 0.390^**^ | | 0.383^**^ | 0.539^**^ | | 0.384^**^ | | 0.431^**^ | | 0.510^**^ | | -0.319^**^ | | -0.288^*^ |
| **Left** | 0.377^**^ | | 0.401^**^ | 0.556^**^ | | 0.399^**^ | | 0.421^**^ | | 0.521^**^ | | -0.292^**^ | | -0.280^*^ |
| **Right** | 0.387^**^ | | 0.352^**^ | 0.502^**^ | | 0.355^**^ | | 0.423^**^ | | 0.479^**^ | | -0.332^**^ | | -0.285^*^ |
| **Dorsolateral Prefrontal Cortex** | |  | | |  | |  | |  | |  | |  | |
| **Left** | 0.397^**^ | | 0.413^**^ | 0.543^**^ | | 0.396^**^ | | 0.455^**^ | | 0.528^**^ | | -0.367^**^ | | -0.236 |
| **Right** | 0.413^**^ | | 0.329^**^ | 0.482^**^ | | 0.289^**^ | | 0.442^**^ | | 0.456^**^ | | -0.378^**^ | | -0.279^*^ |
| **Ventromedial Prefrontal Cortex** | |  | | |  | |  | |  | |  | |  | |
| **Left** | 0.328^**^ | | 0.392^**^ | 0.403^**^ | | 0.265^*^ | | 0.320^**^ | | 0.420^**^ | | -0.265^*^ | | -0.176 |
| **Right** | 0.291^**^ | | 0.281^**^ | 0.335^**^ | | 0.198 | | 0.297^**^ | | 0.335^**^ | | -0.299^**^ | | -0.174 |
| **Orbitofrontal Cortex** | |  | | |  | |  | |  | |  | |  | |
| **Left** | 0.356^**^ | | 0.448^**^ | 0.553^**^ | | 0.450^**^ | | 0.391^**^ | | 0.554^**^ | | -0.299^**^ | | -0.151 |
| **Right** | 0.358^**^ | | 0.377^**^ | 0.504^**^ | | 0.319^**^ | | 0.351^**^ | | 0.477^**^ | | -0.280^*^ | | -0.179 |
| **Motor Cortex** |  | |  |  | |  | |  | |  | |  | |  |
| **Left** | 0.251^*^ | | 0.231^*^ | 0.322^**^ | | 0.226^*^ | | 0.305^**^ | | 0.311^**^ | | -0.243^*^ | | -0.231 |
| **Right** | 0.244^*^ | | 0.173 | 0.272^*^ | | 0.232^*^ | | 0.283^**^ | | 0.275^**^ | | -0.221 | | -0.159 |
| **Total Cingulate Lobe** | 0.301^**^ | | 0.367^**^ | 0.400^**^ | | 0.323^**^ | | 0.355^**^ | | 0.422^**^ | | -0.271^*^ | | -0.254 |
| **Left** | 0.280^**^ | | 0.366^**^ | 0.414^**^ | | 0.347^**^ | | 0.345^**^ | | 0.432^**^ | | -0.252^*^ | | -0.253 |
| **Right** | 0.302^**^ | | 0.341^**^ | 0.354^**^ | | 0.272^*^ | | 0.338^**^ | | 0.380^**^ | | -0.270^*^ | | -0.236 |
| **Anterior Cingulate** |  | |  |  | |  | |  | |  | |  | |  |
| **Left** | 0.270^*^ | | 0.377^**^ | 0.428^**^ | | 0.349^**^ | | 0.336^**^ | | 0.444^**^ | | -0.247^*^ | | -0.228 |
| **Right** | 0.306^**^ | | 0.335^**^ | 0.350^**^ | | 0.269^*^ | | 0.335^**^ | | 0.382^**^ | | -0.295^**^ | | -0.213 |
| **Middle Cingulate** |  | |  |  | |  | |  | |  | |  | |  |
| **Left** | 0.240^*^ | | 0.261^*^ | 0.320^**^ | | 0.277^**^ | | 0.270^*^ | | 0.343^**^ | | -0.174 | | -0.226 |
| **Right** | 0.284^**^ | | 0.238^*^ | 0.319^**^ | | 0.191^*^ | | 0.303^**^ | | 0.316^**^ | | -0.254^*^ | | -0.272^*^ |
| **Posterior Cingulate** |  | |  |  | |  | |  | |  | |  | |  |
| **Left** | 0.244^*^ | | 0.267^*^ | 0.300^**^ | | 0.273^*^ | | 0.295^**^ | | 0.319^**^ | | -0.207 | | -0.244 |
| **Right** | 0.229^*^ | | 0.278^**^ | 0.283^**^ | | 0.217 | | 0.270^*^ | | 0.294^**^ | | -0.160 | | -0.229 |
| **Total Insular Lobe** | 0.292^**^ | | 0.458^**^ | 0.554^**^ | | 0.481^**^ | | 0.359^**^ | | 0.553^**^ | | -0.348^**^ | | -0.156 |
| **Left** | 0.300^**^ | | 0.500^**^ | 0.591^**^ | | 0.532^**^ | | 0.378^**^ | | 0.599^**^ | | -0.373^**^ | | -0.176 |
| **Right** | 0.266^*^ | | 0.391^**^ | 0.485^**^ | | 0.403^**^ | | 0.320^**^ | | 0.477^**^ | | -0.303^**^ | | -0.127 |
| **Anterior Insula** |  | |  |  | |  | |  | |  | |  | |  |
| **Left** | 0.321^**^ | | 0.512^**^ | 0.575^**^ | | 0.503^**^ | | 0.349^**^ | | 0.591^**^ | | -0.357^**^ | | -0.135 |
| **Right** | 0.300^**^ | | 0.410^**^ | 0.497^**^ | | 0.395^**^ | | 0.320^**^ | | 0.498^**^ | | -0.303^**^ | | -0.098 |
| **Posterior Insula** |  | |  |  | |  | |  | |  | |  | |  |
| **Left** | 0.209 | | 0.393^**^ | 0.531^**^ | | 0.509^**^ | | 0.378^**^ | | 0.519^**^ | | -0.346^**^ | | -0.232 |
| **Right** | 0.147 | | 0.279^**^ | 0.376^**^ | | 0.349^**^ | | 0.264^*^ | | 0.348^**^ | | -0.251^*^ | | -0.167 |
| **Total Parietal Lobe** | 0.189 | | 0.203 | 0.288^**^ | | 0.227^*^ | | 0.283^**^ | | 0.273^*^ | | -0.186 | | -0.303^*^ |
| **Left** | 0.193 | | 0.222^*^ | 0.292^**^ | | 0.230^*^ | | 0.274^*^ | | 0.278^**^ | | -0.172 | | -0.294^*^ |
| **Right** | 0.181 | | 0.179 | 0.277^**^ | | 0.218 | | 0.284^**^ | | 0.261^*^ | | -0.195 | | -0.304^*^ |
| **Medial Parietal Cortex** | | |  |  | |  | |  | |  | |  | |  |
| **Left** | 0.141 | | 0.177 | 0.153 | | 0.080 | | 0.206 | | 0.147 | | -0.179 | | -0.293^*^ |
| **Right** | 0.110 | | 0.114 | 0.195 | | 0.112 | | 0.192 | | 0.171 | | -0.195 | | -0.332^**^ |
| **Lateral Parietal Cortex** | | |  |  | |  | |  | |  | |  | |  |
| **Left** | 0.045 | | 0.103 | 0.010 | | 0.033 | | 0.123 | | 0.036 | | -0.131 | | -0.120 |
| **Right** | 0.088 | | 0.067 | 0.060 | | 0.034 | | 0.212 | | 0.072 | | -0.21 | | -0.185 |
| **Sensory Cortex** |  | |  |  | |  | |  | |  | |  | |  |
| **Left** | -0.017 | | 0.026 | -0.101 | | -0.028 | | 0.112 | | -0.029 | | -0.091 | | -0.030 |
| **Right** | -0.110 | | -0.139 | -0.206 | | -0.198 | | -0.008 | | -0.189 | | -0.063 | | 0.001 |
| **Total Temporal Lobe** | 0.196 | | 0.426^**^ | 0.353^**^ | | 0.408^**^ | | 0.325^**^ | | 0.427^**^ | | -0.263^*^ | | -0.197 |
| **Left** | 0.176 | | 0.310^**^ | 0.314^**^ | | 0.418^**^ | | 0.216 | | 0.367^**^ | | -0.177 | | -0.140 |
| **Right** | 0.183 | | 0.299^**^ | 0.244^*^ | | 0.353^**^ | | 0.208 | | 0.324^**^ | | -0.194 | | -0.146 |
| **Dorsolateral Temporal Cortex** | | |  |  | |  | |  | |  | |  | |  |
| **Left** | 0.154 | | 0.410^**^ | 0.332^**^ | | 0.450^**^ | | 0.333^**^ | | 0.419^**^ | | -0.237^*^ | | -0.139 |
| **Right** | 0.145 | | 0.339^**^ | 0.195 | | 0.284^**^ | | 0.272^*^ | | 0.297^**^ | | -0.271^*^ | | -0.137 |
| **Medial Temporal Cortex** | | |  |  | |  | |  | |  | |  | |  |
| **Left** | 0.196 | | 0.410^**^ | 0.404^**^ | | 0.452^**^ | | 0.352^**^ | | 0.469^**^ | | -0.223 | | -0.142 |
| **Right** | 0.158 | | 0.381^**^ | 0.333^**^ | | 0.369^**^ | | 0.289^**^ | | 0.387^**^ | | -0.179 | | -0.089 |
| **Temporal Pole** |  | |  |  | |  | |  | |  | |  | |  |
| **Left** | 0.146 | | 0.382^**^ | 0.376^**^ | | 0.409^**^ | | 0.261^*^ | | 0.411^**^ | | -0.248^*^ | | -0.055 |
| **Right** | 0.149 | | 0.373^**^ | 0.284^*^ | | 0.336^**^ | | 0.237^*^ | | 0.347^**^ | | -0.236 | | -0.065 |
| **Total Occipital Lobe** | 0.135 | | 0.197 | 0.161 | | 0.141 | | 0.228 | | 0.205 | | -0.081 | | -0.227 |
| **Left** | -0.028 | | 0.017 | -0.108 | | -0.044 | | 0.081 | | -0.021 | | 0.027 | | -0.025 |
| **Right** | -0.006 | | 0.052 | -0.044 | | -0.002 | | 0.107 | | 0.027 | | 0.032 | | -0.040 |
| **Hippocampus** |  | |  |  | |  | |  | |  | |  | |  |
| **Left** | 0.192 | | 0.446^**^ | 0.358^**^ | | 0.383^**^ | | 0.276^**^ | | 0.455^**^ | | -0.206 | | -0.179 |
| **Right** | 0.127 | | 0.371^**^ | 0.245^*^ | | 0.276^**^ | | 0.216 | | 0.336^**^ | | -0.152 | | -0.198 |
| **Amygdala** |  | |  |  | |  | |  | |  | |  | |  |
| **Left** | 0.159 | | 0.424^**^ | 0.446^**^ | | 0.473^**^ | | 0.333^**^ | | 0.492^**^ | | -0.282^**^ | | -0.172 |
| **Right** | 0.168 | | 0.480^**^ | 0.408^**^ | | 0.443^**^ | | 0.354^**^ | | 0.483^**^ | | -0.291^**^ | | -0.104 |
| **Caudate** |  | |  |  | |  | |  | |  | |  | |  |
| **Left** | 0.293^**^ | | 0.249^*^ | 0.327^**^ | | 0.259^*^ | | 0.306^**^ | | 0.362^**^ | | -0.158 | | -0.141 |
| **Right** | 0.323^**^ | | 0.198 | 0.299^**^ | | 0.246^*^ | | 0.331^**^ | | 0.344^**^ | | -0.171 | | -0.167 |
| **Putamen** |  | |  |  | |  | |  | |  | |  | |  |
| **Left** | 0.321^**^ | | 0.427^**^ | 0.443^**^ | | 0.406^**^ | | 0.357^**^ | | 0.495^**^ | | -0.313^**^ | | -0.22 |
| **Right** | 0.317^**^ | | 0.382^**^ | 0.379^**^ | | 0.337^**^ | | 0.346^**^ | | 0.438^**^ | | -0.308^**^ | | -0.187 |
| **Accumbens** |  | |  |  | |  | |  | |  | |  | |  |
| **Left** | 0.209 | | 0.361^**^ | 0.372^**^ | | 0.344^**^ | | 0.300^**^ | | 0.404^**^ | | -0.229 | | -0.128 |
| **Right** | 0.239^*^ | | 0.345^**^ | 0.318^**^ | | 0.308^**^ | | 0.306^**^ | | 0.381^**^ | | -0.273^*^ | | -0.060 |
| **Pallidum** |  | |  |  | |  | |  | |  | |  | |  |
| **Left** | 0.225 | | 0.293^**^ | 0.251^*^ | | 0.268^*^ | | 0.253^*^ | | 0.314^**^ | | -0.246^*^ | | -0.136 |
| **Right** | 0.262^*^ | | 0.307^**^ | 0.251^*^ | | 0.251^*^ | | 0.309^**^ | | 0.323^**^ | | -0.299^**^ | | -0.127 |
| **Thalamus** |  | |  |  | |  | |  | |  | |  | |  |
| **Left** | 0.301^**^ | | 0.310^**^ | 0.415^**^ | | 0.349^**^ | | 0.364^**^ | | 0.434^**^ | | -0.243^*^ | | -0.317^*^ |
| **Right** | 0.273^*^ | | 0.271^*^ | 0.350^**^ | | 0.271^*^ | | 0.334^**^ | | 0.370^**^ | | -0.23 | | -0.363^**^ |
| **Total Cerebellum** | 0.232^*^ | | 0.165 | 0.191 | | 0.143 | | 0.314^**^ | | 0.221 | | -0.189 | | -0.174 |
| **Left** | 0.240^*^ | | 0.166 | 0.175 | | 0.135 | | 0.318^**^ | | 0.217 | | -0.185 | | -0.176 |
| **Right** | 0.227^*^ | | 0.166 | 0.211 | | 0.158 | | 0.312^**^ | | 0.229^*^ | | -0.196 | | -0.173 |
| **Vermis** | 0.111 | | 0.049 | 0.014 | | 0.032 | | 0.170 | | 0.065 | | -0.100 | | 0.014 |
| **Pons** | 0.135 | | 0.145 | 0.104 | | 0.065 | | 0.148 | | 0.135 | | -0.148 | | -0.215 |
| **Brainstem** | 0.076 | | 0.001 | -0.049 | | -0.024 | | 0.057 | | 0.001 | | 0.038 | | -0.118 |

ACE = Addenbrooke’s Cognitive Examination; TMT = Trail Making Test. **p* <0.01 ***p* ≤ 0.001

**Supplementary Table 7.** Pearson’s Correlations between Brain Region Volumes and CBI Subdomain scores across all patient groups

|  | **Memory** | **Everyday Skills** | | **Self-Care Skills** | | **Mood** | | **Odd Beliefs** | **Abnormal Behaviour** | | **Eating Habits** | | **Sleep** | | **Stereotypic Behaviour** | | **Reduced Motivation** |
| --- | --- | --- | --- | --- | --- | --- | --- | --- | --- | --- | --- | --- | --- | --- | --- | --- | --- |
| **Total Frontal Lobe** | -0.274^**^ | -0.169 | | 0.111 | | -0.139 | | -0.352^**^ | -0.300^**^ | | -0.353^**^ | | -0.104 | | -0.291^**^ | | -0.310^**^ |
| **Left** | -0.283^**^ | -0.160 | | 0.113 | | -0.171 | | -0.337^**^ | -0.305^**^ | | -0.330^**^ | | -0.117 | | -0.293^**^ | | -0.335^**^ |
| **Right** | -0.254^*^ | -0.172 | | 0.105 | | -0.102 | | -0.354^**^ | -0.284^**^ | | -0.361^**^ | | -0.088 | | -0.279^**^ | | -0.275^**^ |
| **Dorsolateral Prefrontal Cortex** | | |  | |  | |  | | |  | |  | |  | |  | |
| **Left** | -0.238^*^ | -0.217^*^ | | -0.007 | | -0.163 | | -0.313^**^ | -0.308^**^ | | -0.331^**^ | | -0.095 | | -0.205 | | -0.323^**^ |
| **Right** | -0.237^*^ | -0.232^*^ | | -0.024 | | -0.104 | | -0.321^**^ | -0.249^*^ | | -0.365^**^ | | -0.090 | | -0.213^*^ | | -0.264^**^ |
| **Ventromedial Prefrontal Cortex** | | |  | |  | |  | | |  | |  | |  | |  | |
| **Left** | -0.185 | -0.197 | | -0.098 | | -0.101 | | -0.284^**^ | -0.191 | | -0.256* | | 0.016 | | -0.110 | | -0.207 |
| **Right** | -0.172 | -0.157 | | 0.026 | | -0.013 | | -0.278^**^ | -0.2 | | -0.295^**^ | | 0.028 | | -0.142 | | -0.167 |
| **Orbitofrontal Cortex** |  |  | |  | |  | |  |  | |  | |  | |  | |  |
| **Left** | -0.334^**^ | -0.263^**^ | | 0.001 | | -0.196 | | -0.340^**^ | -0.389^**^ | | -0.400^**^ | | -0.061 | | -0.317^**^ | | -0.407^**^ |
| **Right** | -0.330^**^ | -0.235^*^ | | 0.050 | | -0.141 | | -0.365^**^ | -0.342^**^ | | -0.395^**^ | | -0.041 | | -0.299^**^ | | -0.369^**^ |
| **Motor Cortex** |  |  | |  | |  | |  |  | |  | |  | |  | |  |
| **Left** | -0.103 | -0.084 | | 0.096 | | -0.083 | | -0.236^*^ | -0.147 | | -0.163 | | -0.034 | | -0.182 | | -0.104 |
| **Right** | -0.035 | -0.058 | | 0.108 | | -0.024 | | -0.191 | -0.108 | | -0.164 | | 0.006 | | -0.117 | | -0.060 |
| **Total Cingulate** | -0.271^**^ | -0.138 | | 0.111 | | -0.146 | | -0.249^*^ | -0.287^**^ | | -0.247^*^ | | -0.119 | | -0.256^*^ | | -0.297^**^ |
| **Left** | -0.235^*^ | -0.119 | | 0.123 | | -0.136 | | -0.232^*^ | -0.265^**^ | | -0.208 | | -0.070 | | -0.234^*^ | | -0.276^**^ |
| **Right** | -0.292^**^ | -0.148 | | 0.090 | | -0.147 | | -0.249^*^ | -0.290^**^ | | -0.272^**^ | | -0.164 | | -0.262^**^ | | -0.299^**^ |
| **Anterior Cingulate** |  |  | |  | |  | |  |  | |  | |  | |  | |  |
| **Left** | -0.236^*^ | -0.116 | | 0.104 | | -0.130 | | -0.226^*^ | -0.270^**^ | | -0.208 | | -0.060 | | -0.234^*^ | | -0.275^**^ |
| **Right** | -0.283^**^ | -0.149 | | 0.052 | | -0.139 | | -0.213^*^ | -0.290^**^ | | -0.256^*^ | | -0.150 | | -0.239^*^ | | -0.278^**^ |
| **Middle Cingulate** |  |  | |  | |  | |  |  | |  | |  | |  | |  |
| **Left** | -0.129 | -0.060 | | 0.096 | | -0.071 | | -0.072 | -0.148 | | -0.110 | | -0.078 | | -0.133 | | -0.176 |
| **Right** | -0.258^*^ | -0.108 | | 0.111 | | -0.116 | | -0.166 | -0.225^*^ | | -0.209 | | -0.185 | | -0.181 | | -0.255^*^ |
| **Posterior Cingulate** |  |  | |  | |  | |  |  | |  | |  | |  | |  |
| **Left** | -0.182 | -0.101 | | 0.135 | | -0.121 | | -0.195 | -0.2 | | -0.163 | | -0.074 | | -0.186 | | -0.219^*^ |
| **Right** | -0.240^*^ | -0.113 | | 0.141 | | -0.126 | | -0.260^**^ | -0.225^*^ | | -0.238^*^ | | -0.151 | | -0.244^*^ | | -0.269^**^ |
| **Total Insular Lobe** | -0.345^**^ | -0.171 | | 0.077 | | -0.094 | | -0.321^**^ | -0.292^**^ | | -0.346^**^ | | -0.050 | | -0.282^**^ | | -0.324^**^ |
| **Left** | -0.363^**^ | -0.196 | | 0.060 | | -0.111 | | -0.316^**^ | -0.308^**^ | | -0.343^**^ | | -0.044 | | -0.290^**^ | | -0.344^**^ |
| **Right** | -0.308^**^ | -0.137 | | 0.089 | | -0.072 | | -0.306^**^ | -0.260^**^ | | -0.330^**^ | | -0.053 | | -0.257^*^ | | -0.287^**^ |
| **Anterior Insula** |  |  | |  | |  | |  |  | |  | |  | |  | |  |
| **Left** | -0.384^**^ | -0.203 | | 0.037 | | -0.112 | | -0.301^**^ | -0.330^**^ | | -0.376^**^ | | -0.052 | | -0.279^**^ | | -0.373^**^ |
| **Right** | -0.342^**^ | -0.162 | | 0.060 | | -0.090 | | -0.319^**^ | -0.298^**^ | | -0.372^**^ | | -0.066 | | -0.251^*^ | | -0.327^**^ |
| **Posterior Insula** |  |  | |  | |  | |  |  | |  | |  | |  | |  |
| **Left** | -0.260^**^ | -0.151 | | 0.100 | | -0.090 | | -0.299^**^ | -0.21 | | -0.217^*^ | | -0.020 | | -0.267^**^ | | -0.226^*^ |
| **Right** | -0.183 | -0.060 | | 0.137 | | -0.022 | | -0.226^*^ | -0.134 | | -0.184 | | -0.016 | | -0.227^*^ | | -0.151 |
| **Total Parietal Lobe** | -0.106 | -0.034 | | 0.162 | | -0.071 | | -0.259^**^ | -0.128 | | -0.119 | | -0.094 | | -0.181 | | -0.149 |
| **Left** | -0.117 | -0.040 | | 0.149 | | -0.092 | | -0.232^*^ | -0.137 | | -0.117 | | -0.107 | | -0.185 | | -0.173 |
| **Right** | -0.093 | -0.027 | | 0.171 | | -0.049 | | -0.280^**^ | -0.116 | | -0.117 | | -0.080 | | -0.173 | | -0.121 |
| **Medial Parietal Cortex** |  |  | |  | |  | |  |  | |  | |  | |  | |  |
| **Left** | -0.064 | -0.075 | | 0.019 | | -0.019 | | -0.153 | -0.086 | | -0.074 | | -0.096 | | -0.071 | | -0.105 |
| **Right** | -0.056 | -0.036 | | 0.128 | | -0.035 | | -0.228^*^ | -0.112 | | -0.097 | | 0.012 | | -0.130 | | -0.062 |
| **Lateral Parietal Cortex** |  |  | |  | |  | |  |  | |  | |  | |  | |  |
| **Left** | 0.127 | 0.005 | | 0.006 | | 0.045 | | -0.098 | 0.044 | | 0.021 | | 0.098 | | 0.041 | | 0.085 |
| **Right** | 0.090 | 0.007 | | 0.020 | | 0.045 | | -0.159 | 0.063 | | -0.015 | | 0.050 | | 0.042 | | 0.112 |
| **Sensory Cortex** |  |  | |  | |  | |  |  | |  | |  | |  | |  |
| **Left** | 0.17 | 0.065 | | 0.006 | | 0.039 | | -0.044 | 0.090 | | 0.115 | | 0.105 | | 0.082 | | 0.214^*^ |
| **Right** | 0.199 | 0.054 | | -0.027 | | 0.090 | | 0.024 | 0.149 | | 0.110 | | 0.092 | | 0.097 | | 0.193 |
| **Total Temporal Lobe** | -0.232^*^ | -0.051 | | 0.194 | | -0.076 | | -0.196 | -0.240^*^ | | -0.169 | | -0.029 | | -0.264^*^ | | -0.219^*^ |
| **Left** | -0.289^**^ | -0.093 | | 0.149 | | -0.134 | | -0.096 | -0.261^**^ | | -0.166 | | -0.077 | | -0.323^**^ | | -0.293^**^ |
| **Right** | -0.276^**^ | -0.101 | | 0.133 | | -0.099 | | -0.106 | -0.272^**^ | | -0.151 | | -0.097 | | -0.300^**^ | | -0.232^*^ |
| **Dorsolateral Temporal Cortex** | |  | |  | |  | |  |  | |  | |  | |  | |  |
| **Left** | -0.208 | -0.029 | | 0.181 | | -0.056 | | -0.146 | -0.17 | | -0.115 | | 0.033 | | -0.2 | | -0.183 |
| **Right** | -0.197 | -0.064 | | 0.111 | | -0.030 | | -0.176 | -0.189 | | -0.155 | | -0.019 | | -0.201 | | -0.108 |
| **Medial Temporal Lobe** |  |  | |  | |  | |  |  | |  | |  | |  | |  |
| **Left** | -0.252^*^ | -0.132 | | 0.138 | | -0.104 | | -0.282^**^ | -0.233^*^ | | -0.188 | | -0.020 | | -0.279^**^ | | -0.240^*^ |
| **Right** | -0.202 | -0.091 | | 0.132 | | -0.070 | | -0.198 | -0.209 | | -0.120 | | -0.032 | | -0.213 | | -0.162 |
| **Temporal Pole** |  |  | |  | |  | |  |  | |  | |  | |  | |  |
| **Left** | -0.214 | -0.046 | | 0.150 | | -0.023 | | -0.134 | -0.142 | | -0.089 | | 0.028 | | -0.159 | | -0.224^*^ |
| **Right** | -0.224^*^ | -0.004 | | 0.195 | | 0.046 | | -0.122 | -0.150 | | -0.083 | | -0.007 | | -0.137 | | -0.176 |
| **Total Occipital Lobe** | -0.053 | 0.079 | | 0.189 | | -0.060 | | -0.135 | -0.109 | | -0.052 | | -0.024 | | -0.142 | | 0.011 |
| **Left** | 0.169 | 0.197 | | 0.168 | | 0.103 | | 0.008 | 0.088 | | 0.108 | | 0.119 | | 0.096 | | 0.249^*^ |
| **Right** | 0.126 | 0.171 | | 0.173 | | 0.087 | | -0.037 | 0.078 | | 0.083 | | 0.098 | | 0.065 | | 0.21 |
| **Hippocampus** |  |  | |  | |  | |  |  | |  | |  | |  | |  |
| **Left** | -0.346^**^ | -0.151 | | 0.064 | | -0.098 | | -0.314^**^ | -0.244^*^ | | -0.223^*^ | | -0.031 | | -0.293^**^ | | -0.248^*^ |
| **Right** | -0.304^**^ | -0.123 | | 0.077 | | -0.094 | | -0.265^**^ | -0.266^**^ | | -0.201 | | -0.046 | | -0.282^**^ | | -0.184 |
| **Amygdala** |  |  | |  | |  | |  |  | |  | |  | |  | |  |
| **Left** | -0.294^**^ | -0.147 | | 0.098 | | -0.087 | | -0.267^**^ | -0.206 | | -0.205 | | 0.004 | | -0.280^**^ | | -0.203 |
| **Right** | -0.302^**^ | -0.138 | | 0.118 | | -0.093 | | -0.222^*^ | -0.240^*^ | | -0.206 | | 0.014 | | -0.274^**^ | | -0.165 |
| **Caudate** |  |  | |  | |  | |  |  | |  | |  | |  | |  |
| **Left** | -0.043 | -0.062 | | 0.116 | | 0.009 | | -0.212^*^ | -0.142 | | -0.178 | | -0.035 | | -0.144 | | -0.159 |
| **Right** | -0.022 | -0.047 | | 0.146 | | 0.003 | | -0.229^*^ | -0.103 | | -0.164 | | -0.016 | | -0.112 | | -0.113 |
| **Putamen** |  |  | |  | |  | |  |  | |  | |  | |  | |  |
| **Left** | -0.248^*^ | -0.112 | | 0.106 | | -0.010 | | -0.328^**^ | -0.204 | | -0.296^**^ | | -0.038 | | -0.216^*^ | | -0.227^*^ |
| **Right** | -0.191 | -0.131 | | 0.088 | | 0.022 | | -0.355^**^ | -0.181 | | -0.282^**^ | | -0.034 | | -0.185 | | -0.167 |
| **Accumbens** |  |  | |  | |  | |  |  | |  | |  | |  | |  |
| **Left** | -0.253^*^ | -0.130 | | 0.101 | | -0.039 | | -0.279^**^ | -0.213^*^ | | -0.222^*^ | | -0.050 | | -0.249^*^ | | -0.250^*^ |
| **Right** | -0.234^*^ | -0.148 | | 0.075 | | -0.036 | | -0.242^*^ | -0.197 | | -0.18 | | -0.038 | | -0.231^*^ | | -0.188 |
| **Pallidum** |  |  | |  | |  | |  |  | |  | |  | |  | |  |
| **Left** | -0.079 | -0.041 | | 0.069 | | 0.035 | | -0.245^*^ | -0.094 | | -0.166 | | 0.001 | | -0.124 | | -0.065 |
| **Right** | -0.092 | -0.062 | | 0.074 | | 0.070 | | -0.313^**^ | -0.083 | | -0.182 | | -0.007 | | -0.117 | | -0.046 |
| **Thalamus** |  |  | |  | |  | |  |  | |  | |  | |  | |  |
| **Left** | -0.229^*^ | -0.096 | | 0.176 | | -0.118 | | -0.353^**^ | -0.243^*^ | | -0.295^**^ | | -0.047 | | -0.309^**^ | | -0.285^**^ |
| **Right** | -0.207 | -0.107 | | 0.153 | | -0.081 | | -0.349^**^ | -0.208 | | -0.254^*^ | | -0.049 | | -0.288^**^ | | -0.219^*^ |
| **Total Cerebellum** | -0.010 | 0.033 | | 0.171 | | 0.013 | | -0.245^*^ | -0.089 | | -0.136 | | 0.010 | | -0.086 | | -0.044 |
| **Left** | -0.002 | 0.030 | | 0.166 | | 0.017 | | -0.240^*^ | -0.081 | | -0.130 | | 0.000 | | -0.071 | | -0.037 |
| **Right** | -0.031 | 0.034 | | 0.178 | | -0.004 | | -0.245^*^ | -0.110 | | -0.147 | | 0.011 | | -0.110 | | -0.066 |
| **Vermis** | 0.091 | -0.021 | | -0.006 | | 0.060 | | -0.182 | 0.027 | | -0.046 | | 0.046 | | 0.031 | | 0.051 |
| **Pons** | 0.061 | 0.070 | | 0.128 | | 0.153 | | -0.099 | 0.018 | | -0.073 | | 0.095 | | 0.005 | | 0.132 |
| **Brainstem** | 0.2 | 0.088 | | 0.159 | | 0.125 | | -0.055 | 0.111 | | 0.053 | | 0.117 | | 0.048 | | 0.187 |

CBI = Cambridge Behavioral Inventory-Revised **p* <0.01 ***p* ≤0.001
